# Enabling cross-indication protein expression analysis using a curated pan-cancer dataset and a tailored workflow

**DOI:** 10.1038/s41598-026-44872-z

**Published:** 2026-03-23

**Authors:** Jixin Wang, Xiaowen Tian, Wen Yu, Benjamin S. Pullman, John Bullen, Elaine Hurt, Wenyan Zhong

**Affiliations:** 1 Oncology Data Science & AI, AstraZeneca, Gaithersburg, MD USA; 2 Oncology Biometrics, Oncology R&D, AstraZeneca, Gaithersburg, MD USA; 3 Data Science and AI, BioPharmaceuticals R&D, AstraZeneca, Gaithersburg, MD USA; 4 Centre for Genomics Research, Discovery Sciences, R&D, AstraZeneca, Gaithersburg, MD USA; 5 Oncology Targeted Delivery, Research and Early Development, Oncology R&D, AstraZeneca, Gaithersburg, MD USA; 6 Oncology Data Science & AI, Oncology R&D, AstraZeneca, 350 5th Ave, New York, NY 10118 USA

**Keywords:** CPTAC, Proteomics, iBAQ, Normalization, Differential analysis, TCGA, Cancer, Computational biology and bioinformatics

## Abstract

The National Cancer Institute’s Clinical Proteomic Tumor Analysis Consortium (CPTAC) recently generated harmonized genomic, transcriptomic, proteomic, and clinical data for over 1,000 tumors across 10 cohorts to facilitate pan-cancer discovery research. However, protein expression comparison across CPTAC cohorts remains challenging due to non-uniform missing data and varying protein expression distribution patterns across tumor types. To enable the cancer research community to conduct robust cross-cohort protein expression analysis, we present a curated and normalized pan-cancer protein expression dataset derived from the CPTAC pan-cancer study. Our workflow integrates systematic filtering, various missing data handling and normalization strategies. We developed a novel algorithm to select robustly expressed proteins in tumors within any CPTAC cohort; applied a cohort hybrid imputation approach to protein abundance values from FragPipe within each cohort, based on protein expression distribution patterns; and calculated intensity-based absolute quantification using protein abundance values and applied both global and smooth quantile normalization methods. Our analysis demonstrates that global quantile normalization surpasses both smooth quantile normalization and no normalization, as evidenced by its higher rank correlation across cancer cohorts between CPTAC and TCGA for selected proteins. The findings suggest that combining cohort hybrid imputation with global quantile normalization is an effective method for creating a normalized CPTAC pan-cancer protein dataset, which can facilitate the study of protein expression across different cancer types and accelerate cancer research.

**Supplementary Information:**

The online version contains supplementary material available at 10.1038/s41598-026-44872-z.

## Introduction

The identification and prioritization of therapeutic targets are pivotal in both drug development and biological understanding. Traditionally, target prioritization has been predominantly tailored to specific cohorts. However, recent developments in oncology underscore the significance of indication prioritization, where a single target holds relevance across multiple cancer types, thereby streamlining drug development and expediting the creation of novel therapies.

The Clinical Proteomic Tumor Analysis Consortium (CPTAC), established by the National Cancer Institute^1^, stands as a comprehensive resource of global mass spectrometry-based proteomics experiments. These initiatives enable high-throughput biological understanding by elucidating the relative and absolute expression of nearly the entire proteome across over 10 tumor types, inclusive of both tumor tissue and normal adjacent tissue (NAT). The data generated by CPTAC has proven to be instrumental in target discovery and prioritization within tissue cohorts, leveraging a methodology to estimate absolute protein abundance from relative quantitation data^2–4^.

Pan-cancer proteomic efforts across various studies (e.g., ProteomicsDB and Expression Atlas) have been conducted, providing valuable resources in indication assessment^5,6^. However, a substantial challenge remains in comparing protein expression levels across cohorts due to a diverse array of samples, each exhibiting distinct missing data and expression patterns. In this study, we focus on the generation of a standardized, filtered, imputed, and normalized pan-cancer protein expression dataset from the CPTAC resource. Building on a systematic workflow that incorporates evaluation of multiple data processing approaches, we deliver a processed dataset ready for cross-cohort analyses, alongside workflow details to empower researchers in adapting and refining our methodology to suit their specific biological questions. By making this standardized resource available, we aim to enable robust pan-cancer protein expression comparisons and support cancer treatment development and biological insight generation.

## Methods

### Evaluation of CPTAC pan-cancer reprocessed proteomics data

CPTAC pan-cancer samples were reprocessed using the FragPipe computational platform (version 15) with MSFragger (version 3.2)^7^ and Philosopher (version 3.4.13)^8^ for protein identification and quantitation as well as intensity-based absolute quantification (iBAQ) derivation and uniform FragPipe/MSFragger workflow with gene‑level grouping and 1% FDR control, at the fragment and reporter ions level, both the normalization to the pool channel, and the median centering (polishing) can reduce the batch effects due to the sample preparation within the cohort to improve the relative quantitation, and iBAQ/riBAQ to stabilize length‑ and detectability‑related variation as described in Wang et al.^3^ FragPipe was used to assess protein abundance in tumor and normal tissues from 10 cancer indications in TMT labeled CPTAC dataset. Log2-transformed protein abundance was used for assessing missing value patterns and developing an imputation strategy. “Cohort” was defined as the unique combination of tissue type and indication, and “missing rate” was defined as the percentage of samples with missing values in each cohort.

### Algorithm for selection of robustly expressed proteins

An algorithm was developed to select robustly expressed proteins in the samples as follows. For protein *j* in tumor samples from indication *i*, the percentage of samples with missing abundance values was denoted as M_{*ij*} and the median abundance from non-missing observations was denoted as A_{*ij*}. The corresponding percentile rank of A_{*ij*} within each cohort *i* was obtained and denoted as F_*i*{(A_{*ij*})}. The protein *j* was kept in all cohorts if there was an indication *i* such that F_*i*{(A_{*ij*})} > 50% and M_{*ij*} < 25%. In other words, the protein was kept if the median abundance percentile rank was > 50% and the corresponding missing rate was < 25% in at least one indication among tumor samples. The determination of these cutoffs was empirical, aimed at achieving a balance between discarding an excessive number of proteins and retaining those with a high rate of missing protein expression measurements.

### iBAQ conversion and normalization after cohort hybrid imputation

The R package imputeLCMD^9^ was used for imputation after filtering proteins that did not pass the above criteria. This package employs a method selection approach that first classifies the missingness mechanism for each protein^9^. It assumes that the mean protein abundances within the cohort would follow a normal distribution if fully observed. The algorithm estimates the parameters of the complete data distribution through quantile regression on observed feature means. It then sets a censoring threshold by comparing the empirical cumulative distribution function (CDF) of observed data against the theoretical CDF of this estimated complete distribution. A point of maximum positive divergence is determined by computing an objective function designed to quantify the relative difference between these two CDFs, particularly in the lower intensity range. The objective function has the form of $(\frac{{F}_{e}-{F}_{o}+1}{{F}_{o}+1}-1)*10$ where ${F}_{e}$ and ${F}_{o}$ represent the theoretical and empirical CDF, respectively. The intensity value at which this objective function attains its maximal positive value statistically identifies the point of greatest differences of observed cumulative probability compared to the theoretical, thereby establishing the censoring threshold. Proteins with mean observed values at or below this censoring threshold are classified as Missing Not At Random (MNAR), while those above it are designated Missing At Random (MAR) or Missing Completely At Random (MCAR). If the determined missing mechanism was MNAR, quantile regression imputation of left-censored data was applied. Otherwise, k–nearest neighbor (KNN) imputation was applied. Previous studies have shown that KNN imputation performs better in TMT proteomics data^10,11^. Imputation was performed on robustly expressed proteins as determined by the method described above, in log2 scale. iBAQ values were then calculated by dividing the raw imputed protein abundance by the number of theoretically observable peptides of the protein from FragPipe, as described by Wang et al.^3^ The iBAQ values were then normalized using one of two quantile normalization methods: (1) global quantile normalization, with quantile normalization^12^ applied to all samples across indications and tissue types together; or (2) smooth quantile normalization^13^ with the assumption that the statistical distribution of each sample was the same within indications or tissue types, but allowing for differences between groups. Additionally, relative iBAQ (riBAQ)^14^ and riBAQ-derived copy number^15^ were calculated according to Wang et al.^3^ The iBAQ values obtained from the imputed protein abundance was used to derive riBAQ and copy number values without applying additional normalization methods.

### Differential protein expression analysis with normalized iBAQ

To determine whether our missing data imputation and normalization strategy affected downstream analyses, we compared the fold change in differentially expressed proteins (DEP) between tumor and matched NAT samples for each indication, using non-normalized, global quantile-normalized, and smooth quantile-normalized protein iBAQ values.

FragPipe protein abundance–derived iBAQ data from CPTAC tumor and NAT samples were used for DEP analysis using R (version 4.1.1), with empirical Bayes statistics on protein-wise linear models with limma^16^ embedded in the DEP package (version 1.16.0)^17^. The correlations between the log2 fold change from DEP analysis were examined between each comparison using Pearson correlation.

### Comparison of the indication ranks of selected proteins between CPTAC and TCGA

We identified proteins whose protein and RNA expression were highly correlated across CPTAC cohorts prior to normalization. Within each indication, we calculated the Pearson correlation between protein and RNA expression using tumor tissues. Then, proteins with a correlation greater than 0.5 across the majority of indications were kept for subsequent analysis. We then compared their protein expression rank across CPTAC cohorts with their RNA expression rank across corresponding cohorts from The Cancer Genome Atlas (TCGA).^22^ Specifically, the median log2(iBAQ) before and after normalization, the median log2(riBAQ) of CPTAC, and the median log2(TPM) of TCGA were calculated for those proteins within each indication. Next, indications were ranked by the median log2(iBAQ) before and after normalization, the median log2(riBAQ) and the median log2(TPM) of CPTAC and TCGA, respectively. A weighted rank correlation approach^23^ was used to measure rank agreement between each comparison, where the correlation between the protein and RNA expression in CPTAC was used as the weight.

### Sensitivity analysis

To further delineate the impact of imputation and normalization separately, we performed the following sensitivity analyses.

### Comparison of normalization with normalization/imputation impact on analyses for understanding transcriptional differences between tumor and normal tissues

We performed differential expression analysis between tumor and normal tissues for ccRCC and LSCC, using datasets processed through normalization alone as well as normalization combined with imputation. For both indications, we conducted gene ontology enrichment analysis and gene set enrichment analysis (GSEA). Only differentially expressed proteins with fold > = 2 and FDR < = 0.01 were used for gene ontology enrichment and GESA analysis. Gene ontology analysis was performed with enrichR^18^ package and database GO_Biological_Process_2023 was used for query. GSEA analysis was performed with fgsea^19,20^ package, and MSigDB^21^ hallmark 50 gene sets were assessed.

### Subset analysis on global quantile normalization

Global quantile normalization was evaluated using a subset-based approach. After imputation, normalization was applied to all 252 five-cohort subsets constructed from 10 indications. Sample-level concordance was assessed by computing Pearson correlations of normalized log2(iBAQ) values for overlapping samples across five-cohort subsets. Consistency of downstream analyses was assessed by computing log2 tumor-normal fold changes for COAD and LSCC across all relevant five-cohort subsets and calculating pairwise correlations among fold-change vectors. Differential expression analyses were performed within each indication across the relevant five-cohort subsets. Differentially expressed proteins were defined using FDR < 0.01 and absolute fold change > 2, and DEP count distributions were summarized for each indication.

## Results

### Evaluation of CPTAC pan-cancer reprocessed proteomics data

We used the FragPipe computational platform to assess protein abundance in tumor tissue and normal tissue samples from 10 cancer indications in CPTAC. Log2-transformed protein abundance was used to identify missing value patterns and develop an imputation strategy.

**Fig. 1:**
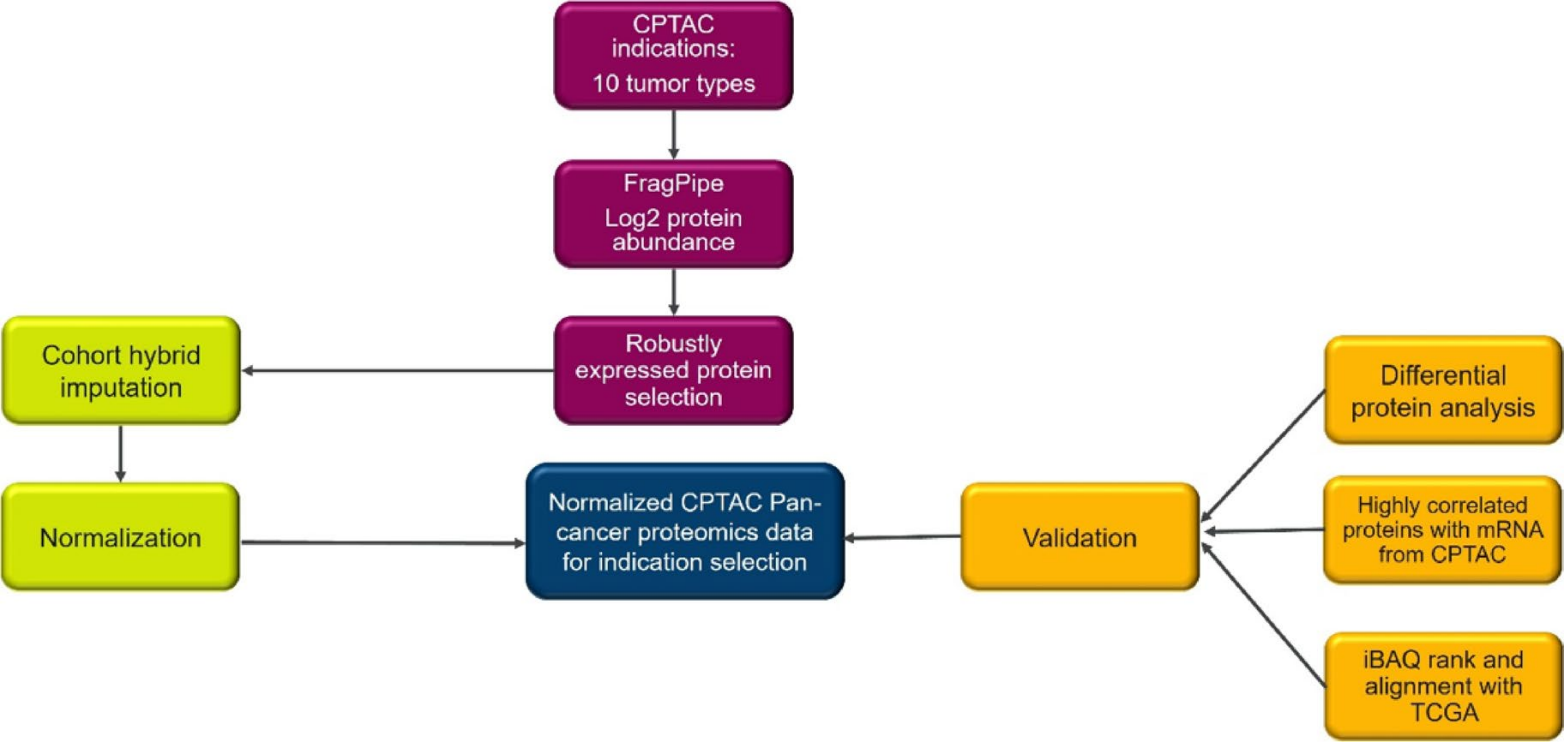
Workflow for a computational approach that enables the comparison of protein expression across CPTAC cohorts. Tissue samples from 10 cancer indications in CPTAC were processed through FragPipe. Protein abundance data were analyzed by iBAQ to derive estimated absolute protein abundance. A cohort hybrid imputation strategy was developed to address missing values. Quantile normalization was applied to iBAQ values. Imputed and normalized iBAQ values were used for validation with differential protein analysis, ranking indications by protein expression and comparing these ranks with those derived from TCGA RNA-Seq data.

The workflow used in this study is outlined in Fig. 1. We developed an algorithm to select robustly expressed proteins across cohorts as described in the Methods section. A total of 15,762 proteins were identified in the union of all cohorts, with 8419 commonly detected proteins across all indications. The protein identifications per cohort at 1% protein-level false discovery rate are shown in Supplementary Table (1). In addition to the 8419 proteins identified in all cohorts, we included 1718 proteins that, while not present in all cohorts, are considered robustly expressed in certain indications by our robustly expressed proteins selection algorithm defined in the Methods section. Finally, we selected 10,137 proteins for further analysis, and the selected proteins per cohort are shown in Supplementary Table (2) Moreover, when we assessed the total iBAQ values for each sample, excluding the filtered proteins versus including them, our algorithm showed a mere 1% reduction (see Supplementary Fig. 1).

### Evaluation of missing values pattern

**Fig. 2:**
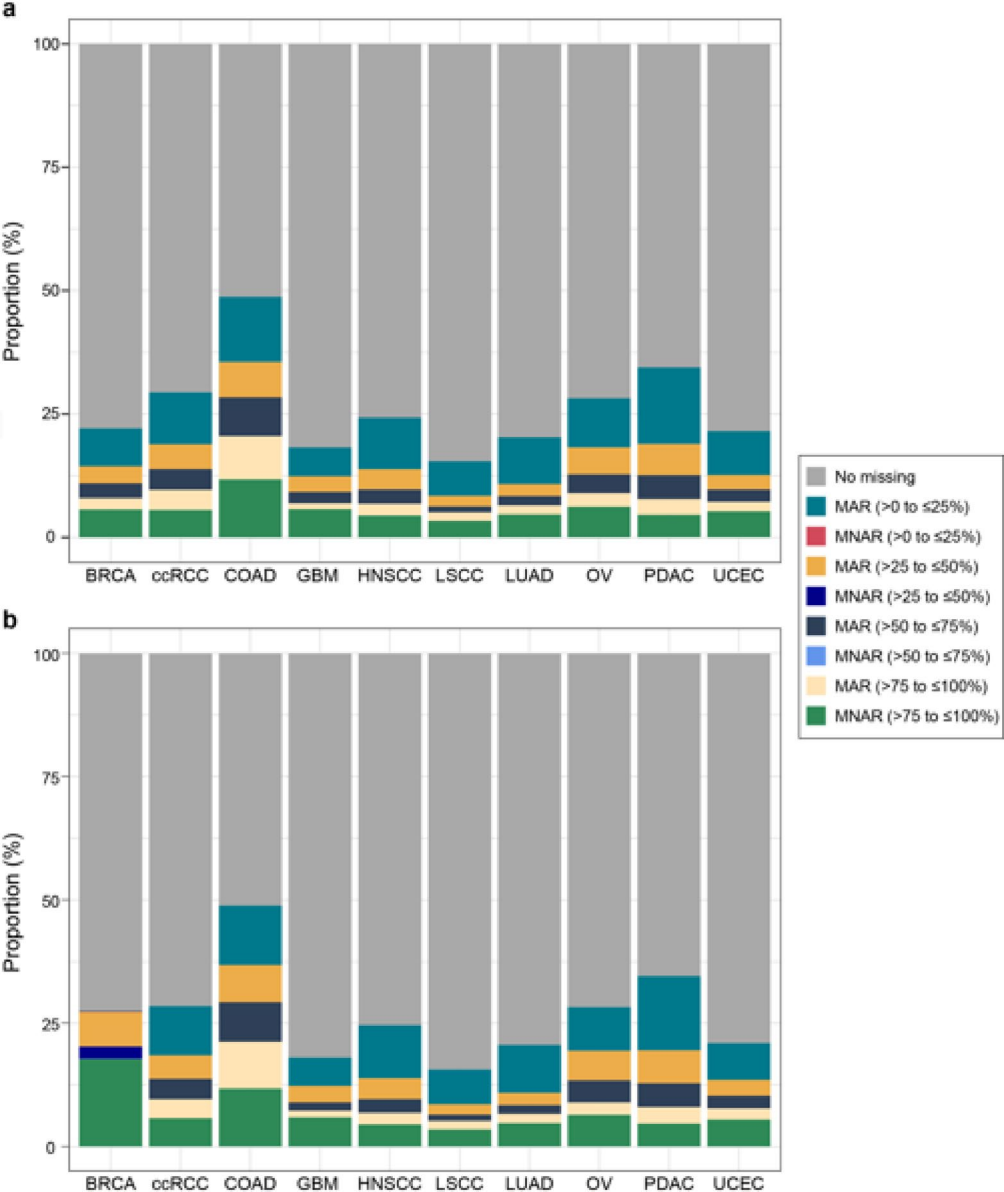
CPTAC missing values pattern assessment in **a** tumor and **b** normal tissue. “Cohort” was defined as the unique combination of tissue type and indication. “Missing rate” was defined as the percentage of samples with missing values in each cohort.

Our evaluation of the missing values showed both missing-at-random (MAR) and MNAR patterns within and across cohorts (Fig. 2a and b). As shown in Fig. 2a, the COAD cohort had the highest missing values among all cohorts. The most prominent categories of proteins with missing values are those with a missing rate of 0–25% in the MAR pattern and those with a missing rate of 75% to 100% in the MNAR pattern. Interestingly, BRCA exhibited a markedly higher number of proteins with the MNAR pattern in normal tissue than in tumor tissue (Fig. 2b). Furthermore, proteins with higher missing rates tended to have lower expression levels (Supplementary Fig. 2), suggesting an MNAR pattern. Because it was critical to apply the appropriate missing value imputation method before performing any further downstream analysis, we chose a cohort hybrid algorithm to impute missing values^9^.

### Assessment of normalization methods

**Fig. 3:**
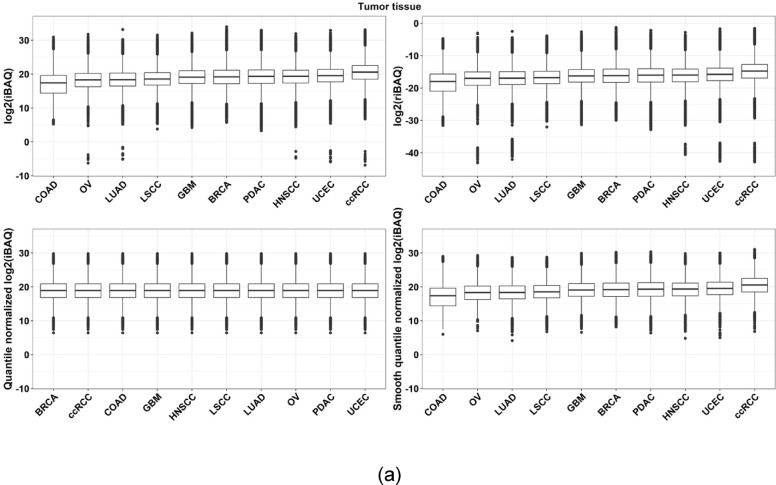

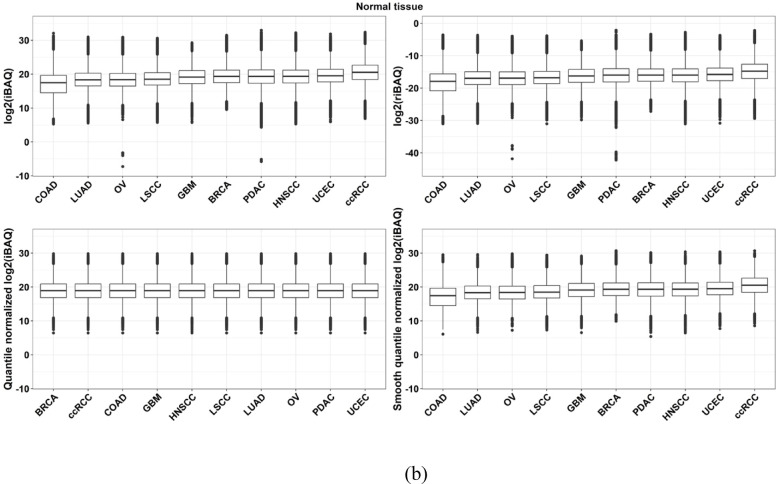
Quantile normalization. **a** Quantile normalization applied to derived iBAQ values after imputation in **a** tumor and **b** normal tissue. Two quantile normalization methods, global and smooth quantile normalization, were used

Global quantile normalization ensured identical distribution across cohorts for both tumor and normal tissues, whereas smooth quantile normalization preserved variability across cohorts (Fig. 3a and b). The principal component analysis (PCA) analysis of protein expression across pan-cancer indications revealed that cohort separation based on protein expression was more distinct than that of tissue types, both before and after normalization. The most noticeable tissue separation occurred following global quantile normalization (Supplementary Fig. 3 and Supplementary Fig. 4).

### Differential protein expression analysis with normalized and imputed iBAQ values

**Fig. 4:**
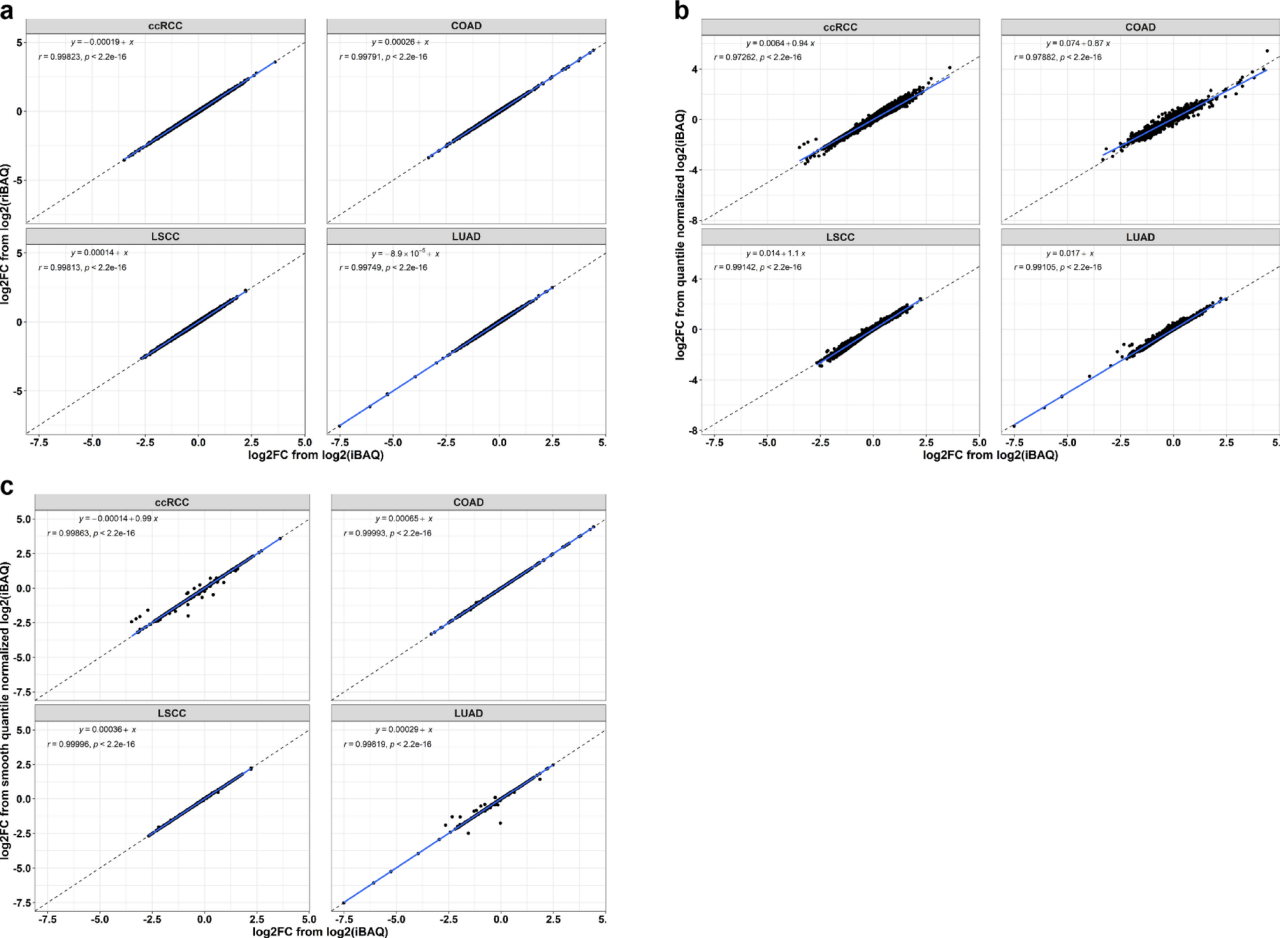
Correlation between fold change from differential expression analysis. **a** riBAQ normalization and no normalization. **b** Global quantile normalization and no normalization. **c** Smooth quantile normalization and no normalization.

To determine whether our missing data imputation and normalization strategy affected downstream analyses, we compared the fold change in DEP between tumor tissue and matched normal tissue of selected cohorts (ccRCC, COAD, LSCC, and LUAD), using non-normalized, riBAQ, global quantile normalized, and smooth quantile normalized protein iBAQ values. Our results (Pearson *r* values) demonstrate a strong correlation in fold change between riBAQ normalized data and non-normalized data (ccRCC, *r* = 0.99823; COAD, *r* = 0.99791; LUAD, *r* = 0.99749; LSCC, *r* = 0.99813) (Fig. 4a), and global quantile normalized data and non-normalized data (ccRCC, *r* = 0.97262; COAD, *r* = 0.97882; LUAD, *r* = 0.99105; LSCC, *r* = 0.99142) (Fig. 4b). Similar results were observed when we compared smooth quantile normalized data with non-normalized data (ccRCC, *r* = 0.99863; COAD, *r* = 0.99993; LUAD, *r* = 0.99819; LSCC, *r* = 0.99996) (Fig. 4c). The Pearson *r* values tended to decrease as more variability was removed from the dataset; for example, the smooth quantile normalization resulted in *r* = 0.99863, whereas the global quantile normalized resulted in *r* = 0.97262 in ccRCC. However, with global quantile normalization, the correlations were still greater than 0.97 in the four selected indications, indicating that the global quantile normalization method retained biological differences between tumor tissues and matched normal tissues within cohorts. The estimated log2-fold change, p-value, and p-values adjusted for multiple testing using Benjamini-Hochberg procedure^24^ (FDR) are presented in Supplementary Tables S3-S6.

To benchmark the impact of imputation and quantile normalization, we assessed changes in DEPs identified in LUAD cohort. Applying a threshold of log2-fold change > 1 and FDR < 0.01, we compared DEPs identified using different versions of data: raw data, imputed data where only missing not at random (MNAR) values are imputed, imputed data where both MNAR and missing at random (MAR) values are imputed, imputation of MNAR followed by quantile normalization, and imputation of both MNAR and MAR followed by quantile normalization (Supplementary Table S7). Prior to normalization, imputing MAR values in addition to MNAR resulted in the identification of 23 additional DEPs. However, the power increase from imputing MAR is limited: of the 23 additional DEPs found after KNN imputation, 10 have a log2-fold change above 0.8 prior to KNN imputation is applied. As expected, more extensive data processing leads to a reduction in the overlap of DEPs shared between raw data and processed datasets, dropping from 98.1% (raw versus MAR imputation only) to 75.8% (raw versus full imputation with quantile normalization) as shown in supplementary Fig. 5. Among the 67 DEPs identified exclusively after imputation and normalization (relative to raw data), 52 (78%) still showed a log2-fold change greater than 0.8 in the raw dataset (Supplementary Fig. 5). However, we observed that the imputed dataset yielded four DEPs with notably large log2-fold change values (ranging from − 3.7 to -7.7). Therefore, external validation is necessary to distinguish whether these observations reflect true biological signals or artifacts of the imputation process. While the overarching goal of cross-indication analysis necessitates retaining all proteins, it is particularly important to interpret results for cohorts with completely missing protein abundances with caution.

### Comparison of the indication ranks of selected proteins between CPTAC and TCGA

We identified proteins whose protein and RNA expression were highly correlated (*r* > 0.5) across CPTAC cohorts: ERAP2, CA9, GSTM3, MX1, and STAT1. We then compared their median protein expression rank across CPTAC cohorts with their median RNA expression rank across corresponding TCGA cohorts. A weighted rank correlation approach^23^ was used to measure rank agreement between each comparison (e.g., denoted as “v” in Supplementary Fig. 5). OV and glioblastoma multiforme GBM were excluded from the weighted rank correlation calculation because they had a small number of proteins with *r* > 0.5, and those proteins did not overlap with those selected from other indications. Global quantile normalization had a higher rank correlation (weighted rank correlation of 0.597–0.931) than smooth quantile normalization (weighted rank correlation of 0.168–0.76) or no normalization (weighted rank correlation of 0.168–0.76) (Supplementary Fig. 8).

### Comparison of normalization with normalization/imputation impact on analyses for understanding transcriptional differences between tumor and normal tissues

Differential expression analysis results between tumor and normal tissues for both ccRCC and LSCC, using datasets processed through normalization alone as well as normalization combined with imputation. Results from the comprehensive abundance analyses covering both normalization +imputation and normalization-only approaches are available in Supplementary Tables 8–11.

For ccRCC and LUAD indications, gene ontology enrichment analysis identified highly similar top biological process terms from the normalized+imputed datasets and the normalization-only datasets (Supplementary Fig. 6 and Supplementary Fig. 7). Likewise, GSEA highlighted comparable MSigDB signaling pathways as enriched in both data processing approaches (Supplementary Fig. 6 and Supplementary Fig. 7). At an FDR threshold of 0.01, there are 223 overlapping biological processes between the normalized/imputed and normalization-only datasets for ccRCC. For LSCC, 161 biological processes are overlapped at the same threshold (Supplementary Fig. 6 and Supplementary Fig. 7). These results suggest that imputation has minimal impact on the identification of relevant biological processes, indicating that our analysis workflow preserves the underlying biology while enabling comparisons across indications.

### Subset analysis on global quantile normalization

To further assess the potential impact of global quantile normalization on biological variation, we applied the normalization procedure to all 252 possible 5-cohort subsets derived from the 10 available indications after imputation. The consistency of normalized data resulting from different subsets was examined in two ways.

First, we assessed the correlations between normalized log2(iBAQ) values for overlapping samples present in different pairs of 5-cohort subsets. For each pair of 5-cohort subsets, we identified samples that appeared in both subsets and compared their log2(iBAQ) values. Due to computational constraints, we did not evaluate all possible pairs of subsets; however, a representative analysis using samples from cohorts (BRCA, ccRCC, COAD, GBM, HNSCC), paired with all possible 5-cohort subsets, showed a minimum Pearson correlation coefficient of 0.9987. The high level of agreement indicates substantial stability of the normalization process and suggests that downstream analyses based on this normalized data will be highly consistent.

Second, we evaluated the consistency of downstream analyses by comparing fold changes between tumor and matched normal tissues in COAD and LSCC across all relevant 5-cohort subsets. There are 126 possible of 5-cohort subsets that include samples from COAD (or LSCC), and each subset generates a set of fold change estimates. We calculated the pairwise correlations among these log2-transformed fold changes and visualized using heatmaps (Supplementary Fig. 9). As expected, the heatmaps demonstrated a consistently high level of agreement in fold change estimates across subsets, further supporting the consistency of the downstream analyses.

Additionally, we have conducted differential expression analysis between tumor and normal tissues using 5-cohort subsets. Specifically, for each indication, we considered all relevant 5-cohort subset combinations (126 subsets per indication), allowing us to quantify the number of DEPs identified in each case. DEPs were defined consistently using the same criteria (FDR < 0.01 and absolute fold change > 2). We have summarized the distribution of DEP counts for each indication in supplementary Fig. 10. For most indications, the number of DEPs remains relatively stable across different 5-cohort subsets, suggesting a consistent effect of data processing strategy. We observed notably greater variability in ccRCC, LSCC, and UCEC, with interquartile ranges of 61, 71, and 101, respectively. This increased variability may be attributable to quantile normalization moderately compressing or enlarging biological effects in these indications, subsequently affecting the number of DEPs detected.

Taken together, these results suggest that global quantile normalization can substantially minimize technical variation while maintaining underlying biological differences, indicating that the principal biological conclusions are generally robust to the specific composition of cohorts included in the normalization.

## Discussion

CPTAC recently generated harmonized genomic, transcriptomic, proteomic, and clinical data for over 1000 tumors in 10 cohorts to facilitate pan-cancer discovery research^2^. To use these data for prioritizing cancer surface antigens and selecting indications in the discovery and development of drug targets, protein expression levels often need to be compared and ranked across cohorts^25,26^. Efforts to do so are hindered, however, by non-uniform missing data and varying protein expression distribution patterns across tumor types. To evaluate various strategies for missing data handling and normalization for the generation of a normalized pan-cancer protein expression dataset, we built a computational workflow that incorporates the selection of robustly expressed proteins, cohort hybrid imputation, and quantile normalization^27^. We then evaluated our strategy by comparing indication ranking between CPTAC and TCGA, using a set of proteins with high correlation between protein and RNA expression (Fig. 1). To our knowledge, this work represents the first cross-cohort normalized CPTAC pan-cancer data containing estimated absolute protein expression quantification derived from mass spectrometry–based proteomic data.

There are considerable challenges in harmonizing proteomics data across the 10 CPTAC cohorts because they have been generated over multiple years and analyzed by various laboratories using different processing pipelines. To avoid potential confounding factors, it was necessary to reanalyze TMT global proteomics data with the same pipeline. We first estimated the absolute protein abundance in reprocessed CPTAC pan-cancer data as described previously^3^. In addition, to create a unified dataset, it was necessary to define a set of proteins that maximizes the identification of proteins across all 10 cohorts with high quality while minimizing the occurrence of missing values. We designed a protein selection algorithm to effectively balance these requirements. Our robustly expressed proteins selection algorithm defined 10,137 proteins, which indicated that more than half of the human proteome (assuming 20,000 protein products)^28,29^ is robustly expressed.

Proteomics data often contain missing values due to a variety of technical factors, such as protein abundance below the instrument limit of detection, poor ionization efficiency, and low signal-to-noise ratio^30–32^. When merging the 10 cohorts, we faced similar issues. Although many algorithms have been developed for missing value imputation^33^, it is critical to select the ones that are best suited to the specific characteristics of the data and downstream analysis needs. An analysis of the missing values in the CPTAC cohort uncovered patterns indicative of both MAR and MNAR. Based on these observations, a cohort-hybrid imputation approach was applied. Our study introduces a general framework to manage missing data when comparing protein expression across cohorts. For other specific analyses, one can adopt this model, integrating multiple imputation methods to measure the uncertainty linked to imputation, thereby obtaining more accurate estimations.

Due to the lack of a definitive list of proteins with known expression levels across different tumor types, we chose to use proteins that exhibit a strong correlation between their RNA and protein expression levels. We then assessed our approach by comparing the rank order of these proteins’ expression in our normalized dataset to their RNA expression rank in TCGA as an indirect method of evaluation. Furthermore, we implemented a weighted rank correlation method that accounts for the correlation between protein and RNA expression levels. The results of our validation method showed a significantly enhanced rank correlation with the application of global cohort normalization as opposed to using a dataset without normalization (see Supplementary Fig. 8). Further, when comparing the fold change of tumor versus matched normal tissues using the normalized dataset versus the original dataset, there was significant agreement (Fig. 4), indicating that our normalization method did not inadvertently eliminate biological variation.

Our assessment indicates that a combination of cohort hybrid imputation and global quantile normalization is a reasonable approach to generate a normalized CPTAC pan-cancer protein dataset that could be leveraged to compare protein expression across different cancer types. Nonetheless, the limited number of proteins and indications used for validation in our study represents an important limitation. While our approach was tested using selected highly correlated proteins and rank alignment with TCGA RNA-Seq data, we recommend further validation using a larger number of proteins with expression levels measured across cohorts with other orthogonal experimental methods such as targeted proteomics to enhance the robustness of our approach.

Quantile normalization assumes that, across runs or samples, the majority of proteins are drawn from the same underlying distribution—i.e., most proteins are unchanged and present at comparable concentrations—so observed global differences primarily reflect technical rather than biological effects. In a pan‑cancer, multi‑tissue, multi‑cohort TMT design, this assumption can be challenged because tumor types and adjacent normal tissues often exhibit genuine global proteomic shifts^34,35^. Our preprocessing is not intended to bypass this assumption; it mitigates known technical heterogeneity before cross‑cohort harmonization.

We have established a computational workflow for cross-indication comparison from proteomics data, thereby providing a unique data resource to interrogate protein expression across different cancer types. Despite our efforts to normalize the CPTAC pan-cancer protein dataset across different cancer cohorts, batch effects may still persist due to upstream technical variations, such as differences in laboratory practices and platforms^36^. Our results demonstrate that missing data imputation and normalization strategies do not affect downstream analyses. The methodology and the data sources used in this study can serve as valuable resources for cancer research. Despite evaluating multiple normalization strategies (including global quantile normalization, smooth quantile normalization, and riBAQ), we did not implement median normalization in the primary analysis. We recognize that this more comprehensive normalization can result in greater alteration to the original data structure, which may affect subtle biological signals. To address this concern, we conducted downstream sensitivity analyses demonstrating that key biological patterns and signals remain preserved following quantile normalization. A comprehensive benchmarking of all potential normalization strategies, including alternative approaches beyond those implemented here, remains an important direction for future research and will be critical for optimizing data integration and downstream analyses in large proteomics studies.

## Supplementary Information

Below is the link to the electronic supplementary material.

Supplementary Material 1

Supplementary Material 2

Supplementary Material 3

Supplementary Material 4

Supplementary Material 5

Supplementary Material 6

Supplementary Material 7

Supplementary Material 8

Supplementary Material 9

Supplementary Material 10

## Data Availability

The search output and reports from the FragPipe, Protein‑level data without normalization and without imputation, Protein‑level data after imputation (no normalization) and protein‑level data for each normalization method after imputation of protein abundance estimation for the 10 CPTAC indications used in this work and scripts can be downloaded from https://doi.org/10.5281/zenodo.17203216.
