# Supplementary material for: Enabling cross-indication protein expression analysis using a curated pan-cancer dataset and a tailored workflow
Source: Sci Rep. 2026 Mar 23;16:14623. doi: 10.1038/s41598-026-44872-z (PMC13153371; doi:10.1038/s41598-026-44872-z)
Supplement: Supplementary file 10 — Supplementary Material 10 [file 41598_2026_44872_MOESM10_ESM.docx]

**Enabling cross-indication protein expression analysis using a curated pan-cancer dataset and a tailored workflow**

Jixin Wang*, Xiaowen Tian*, Wen Yu, Benjamin S Pullman, John Bullen, Jr.,

Elaine Hurt, Wenyan Zhong

**Supplementary Information**


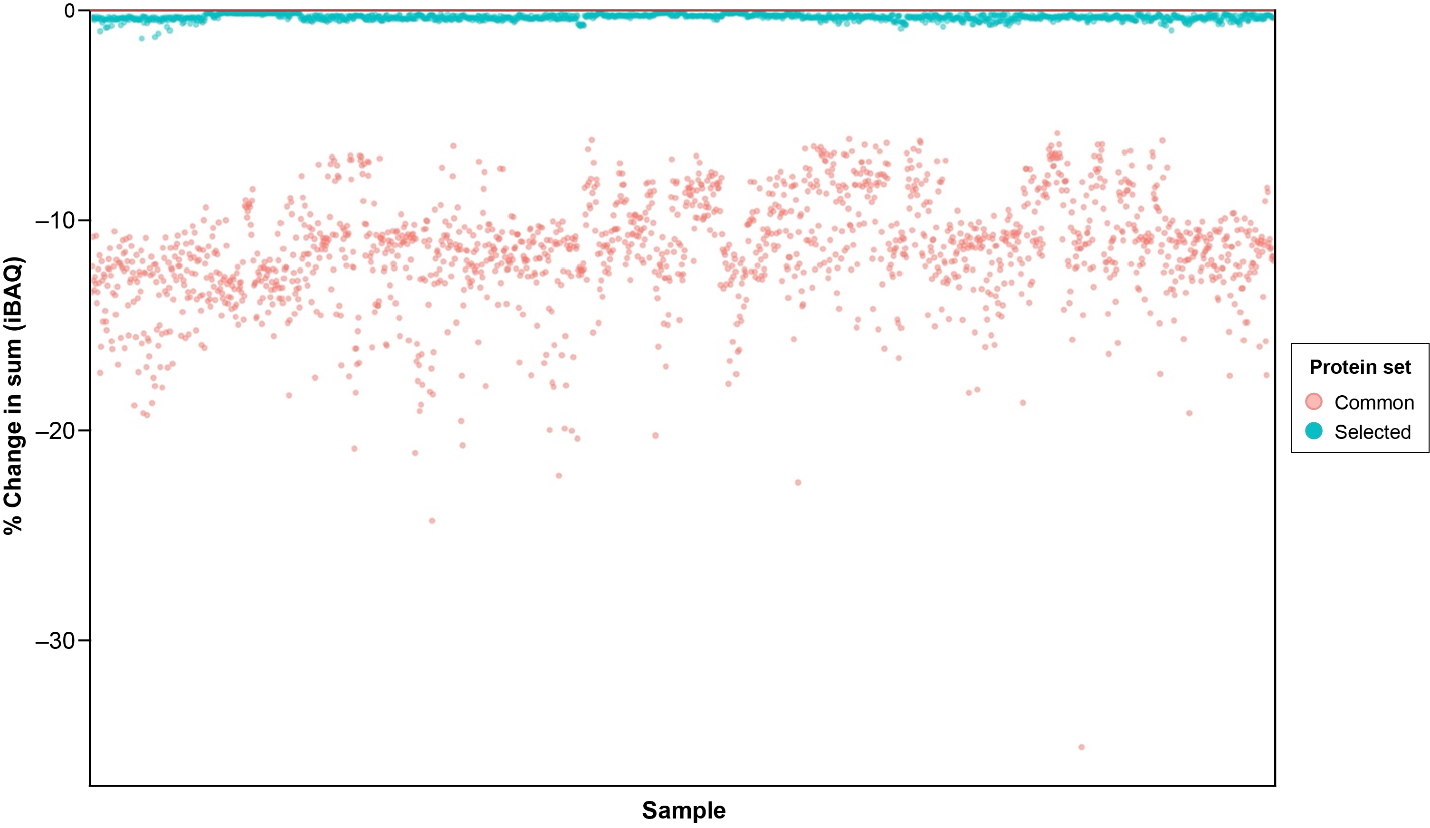


**Supplementary Figure 1.** Percentage change in the sum of intensity-based absolute quantification (iBAQ) values for each sample. “Common,” proteins commonly detected across all indications; “Selected,” robustly selected proteins.


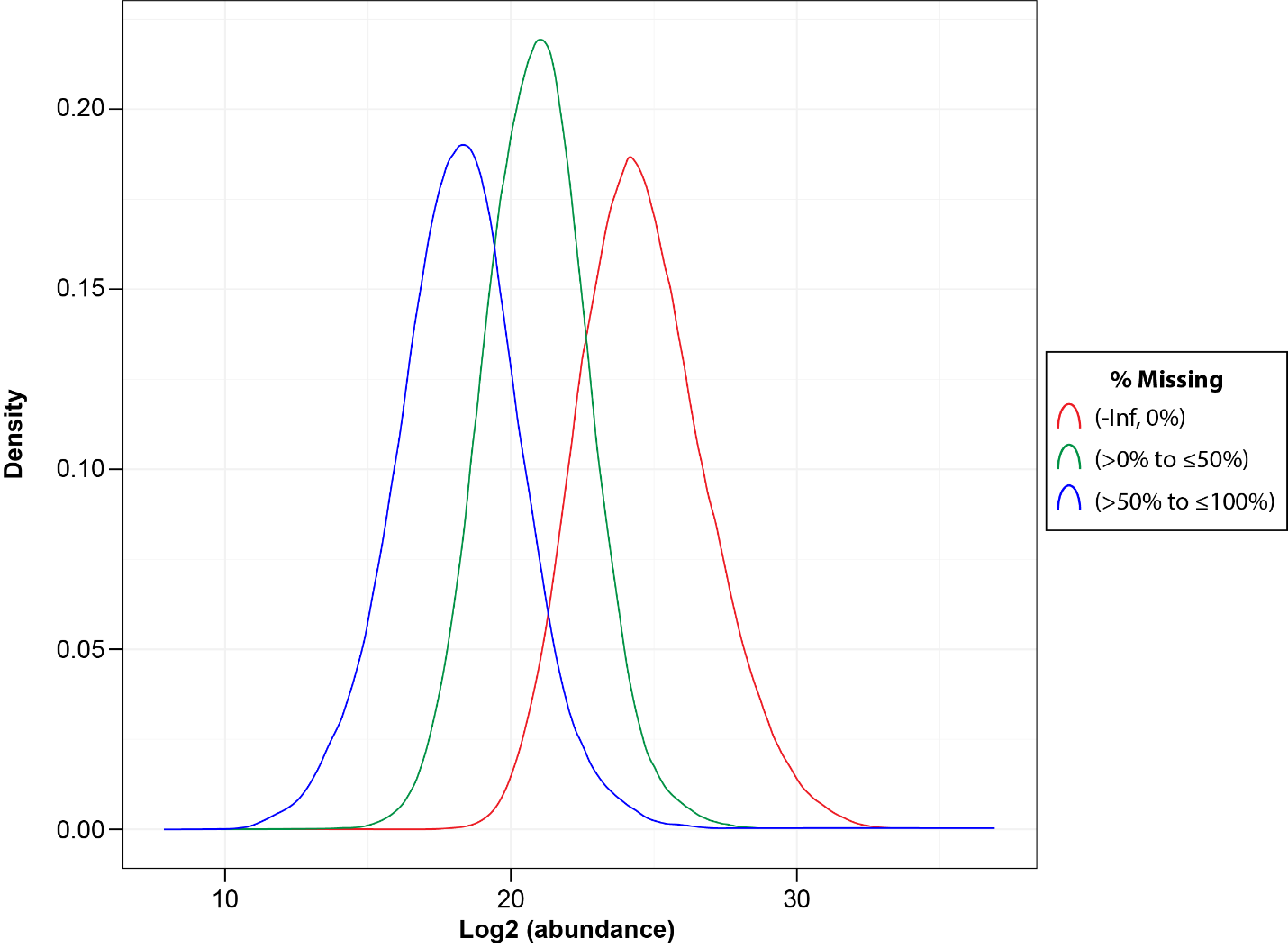


**Supplementary Figure 2.** Protein expression missing pattern in the National Cancer Institute’s Clinical Proteomic Tumor Analysis Consortium (CPTAC) dataset.


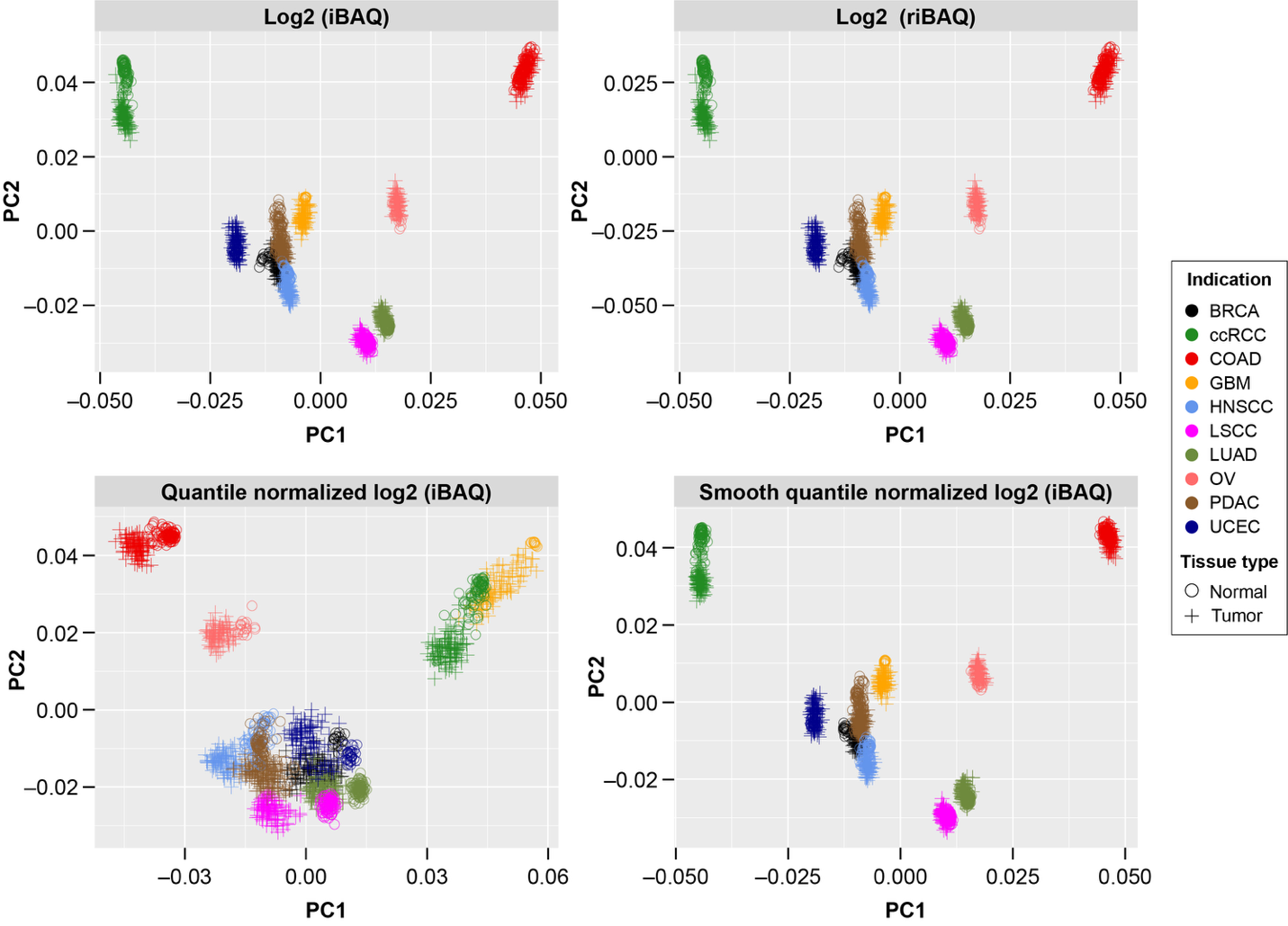


**Supplementary Figure 3.** Principal component analysis of protein expression across pan-cancer indications in the CPTAC dataset. Data points are color coded by indication and shaped according to tissue type. BRCA, breast cancer; ccRCC, clear-cell renal cell carcinoma; COAD, colon adenocarcinoma; GBM, glioblastoma multiforme; HNSCC, head and neck squamous-cell carcinoma; LSCC, lung squamous-cell carcinoma; LUAD, lung adenocarcinoma; OV, ovarian cancer; PDAC, pancreatic ductal adenocarcinoma; UCEC, uterine corpus endometrial carcinoma.

a


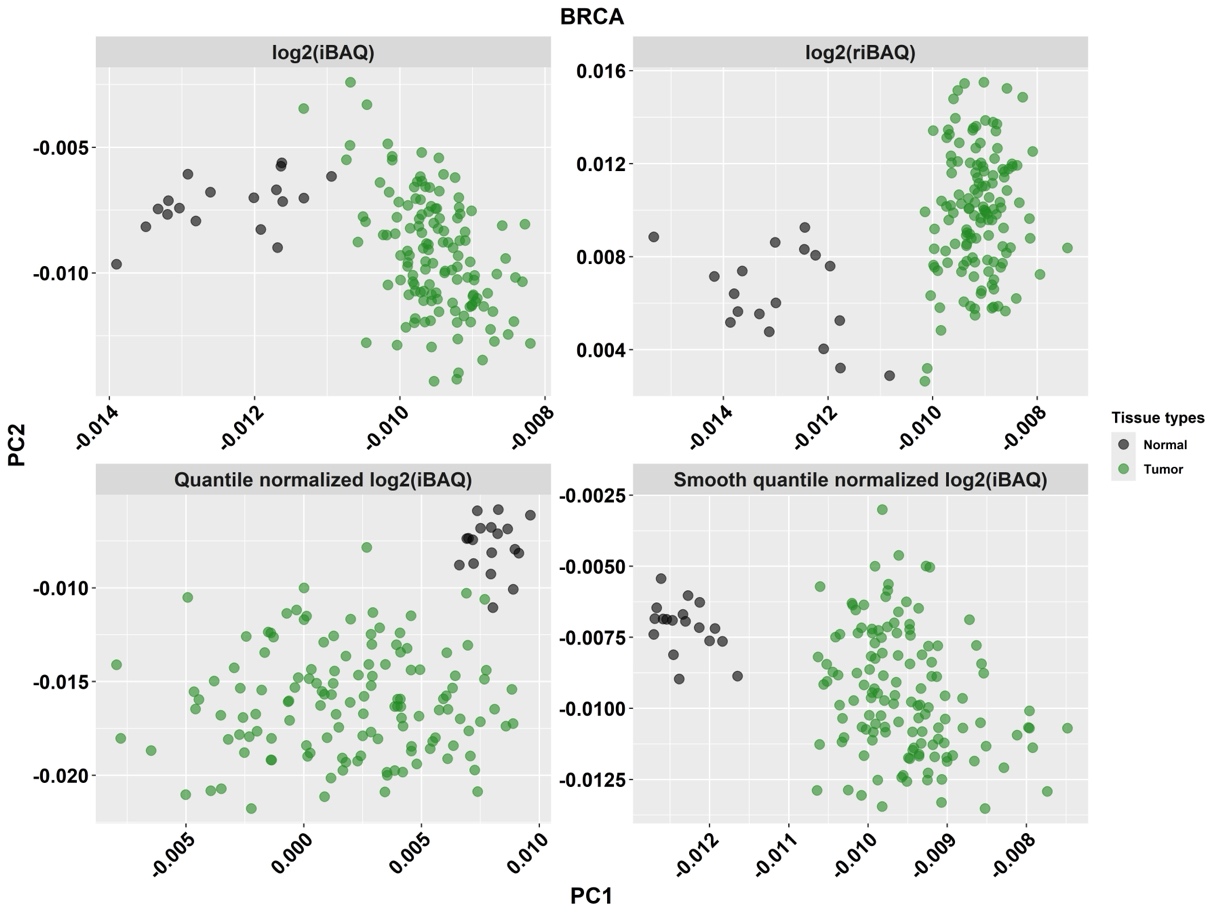


b


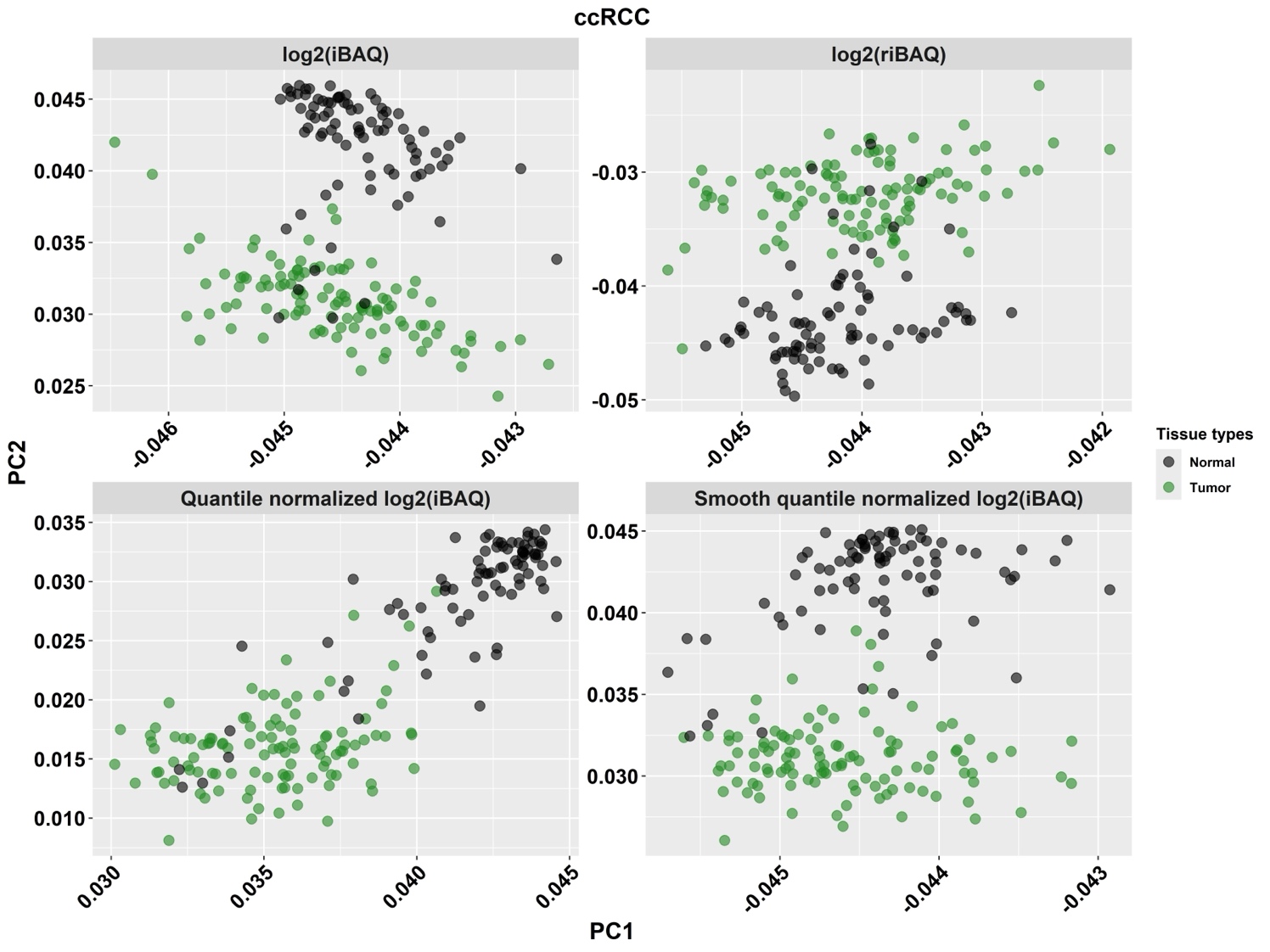


c


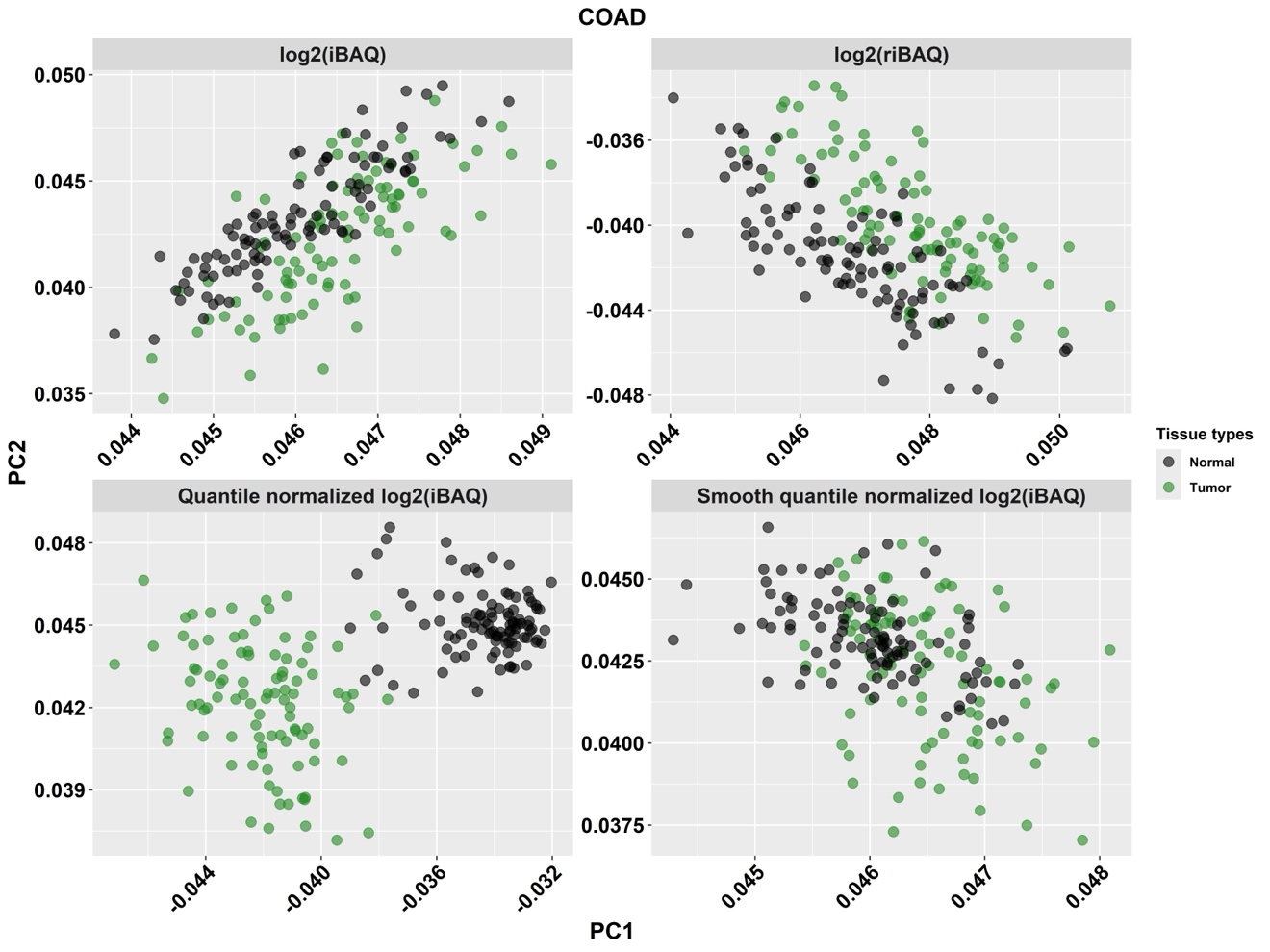


d


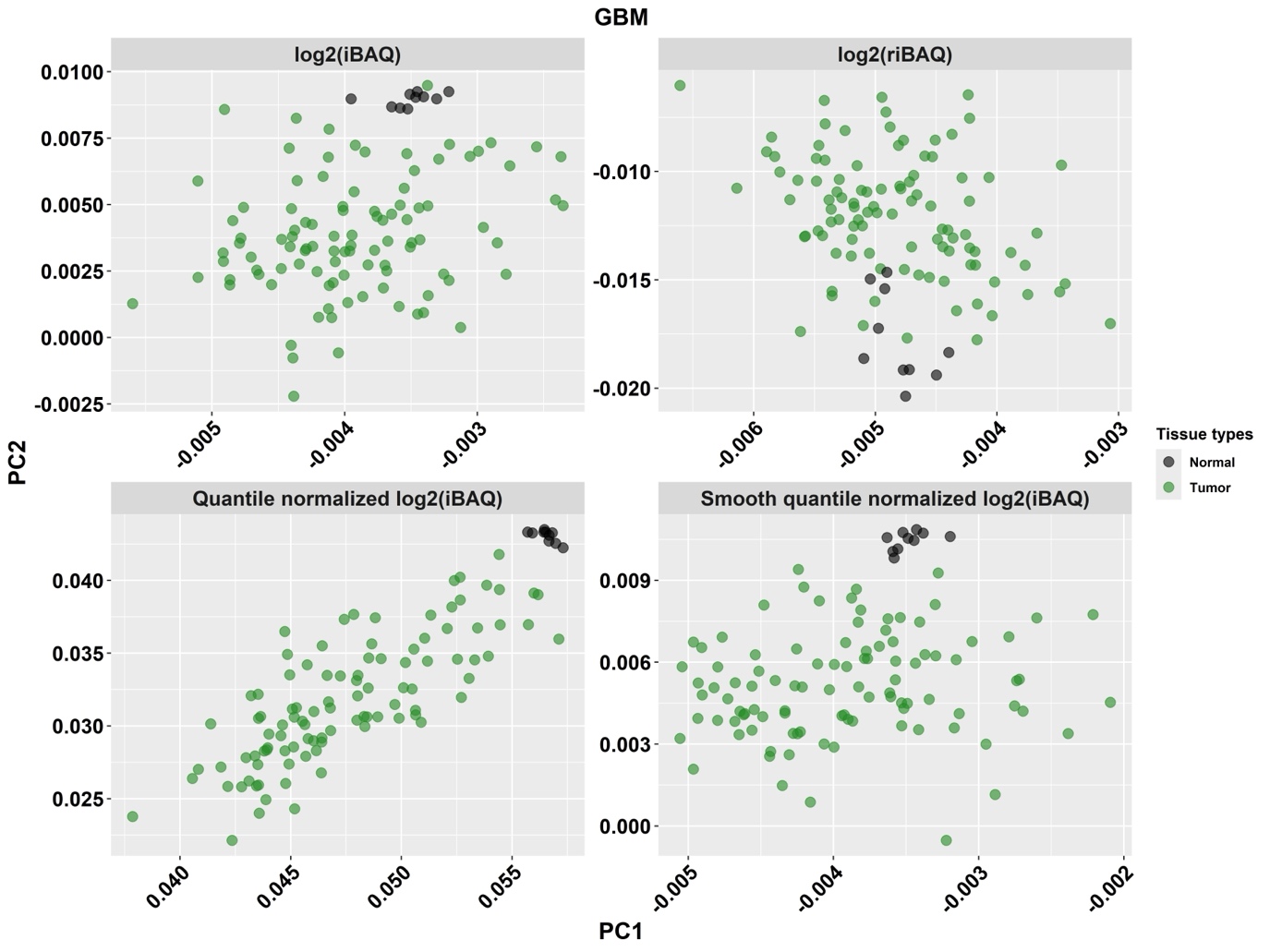


e


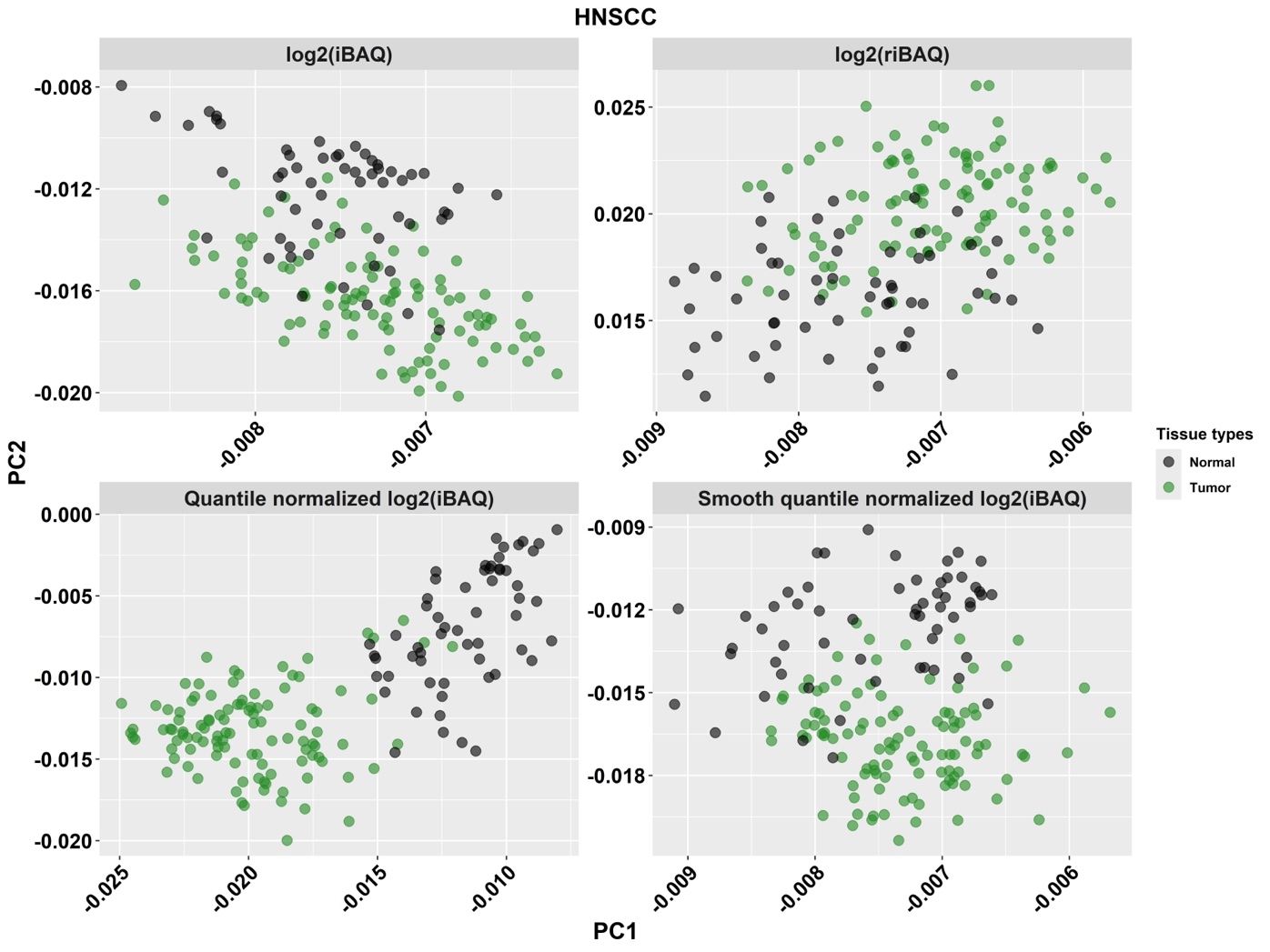


f


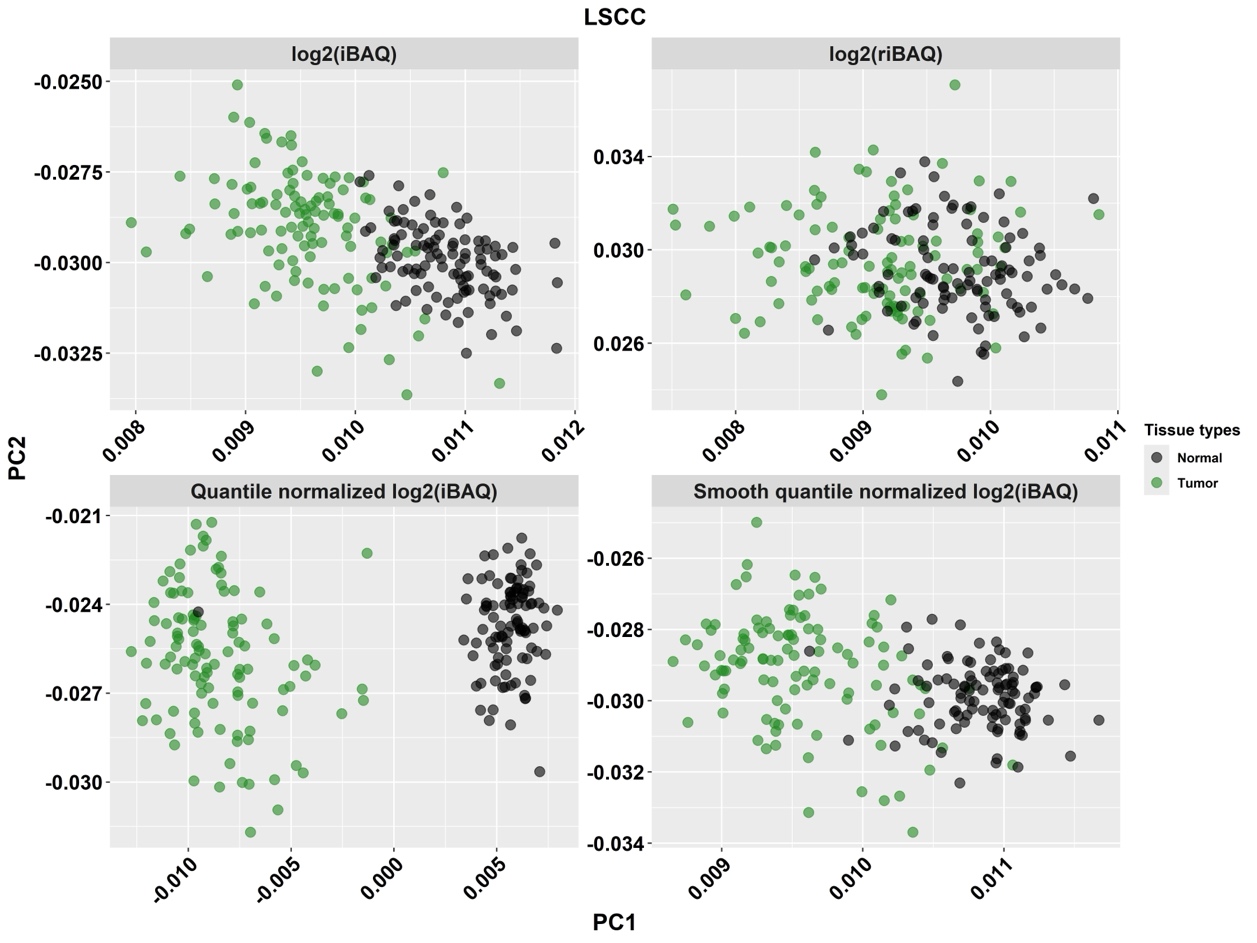


g


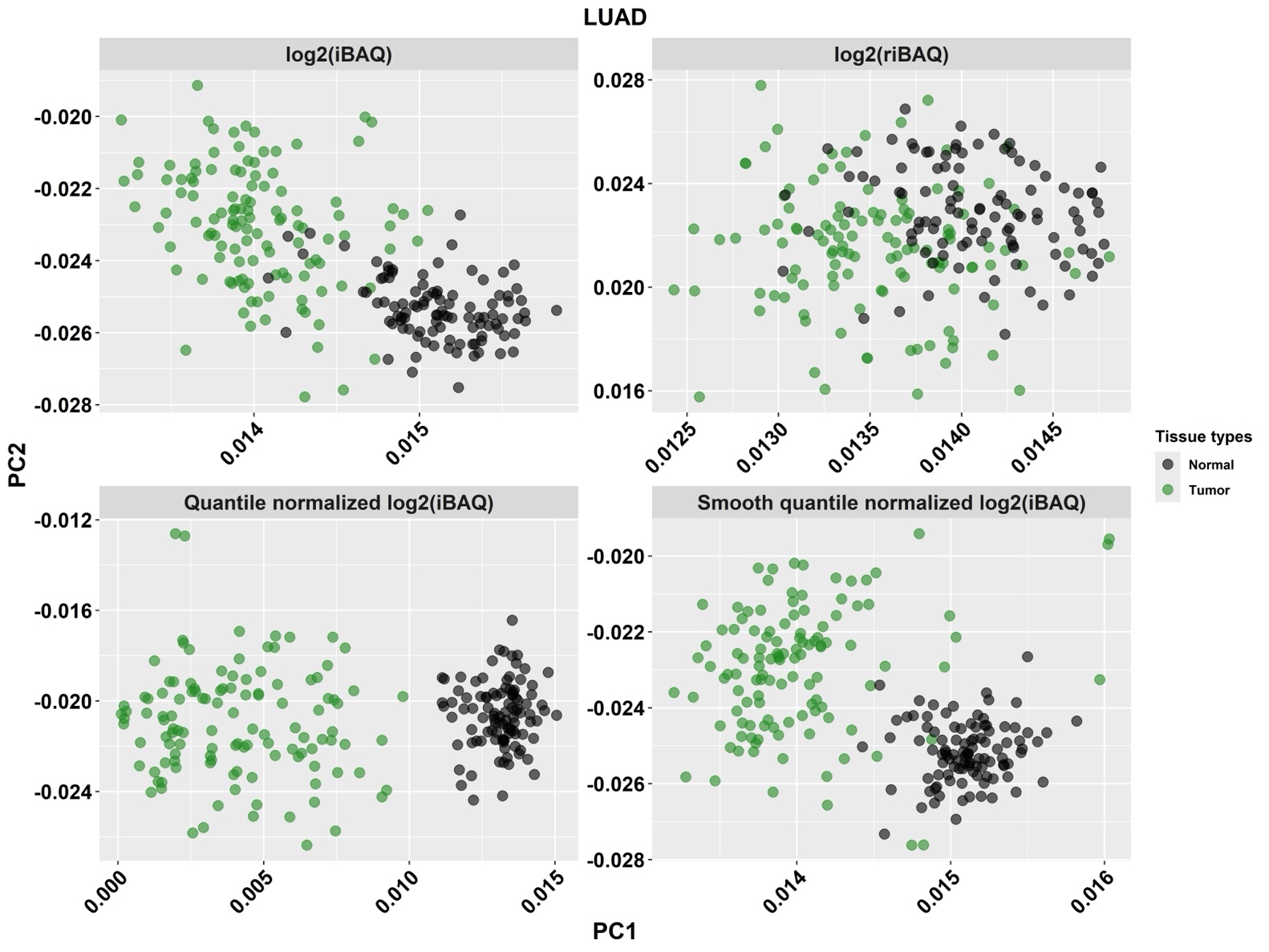


h


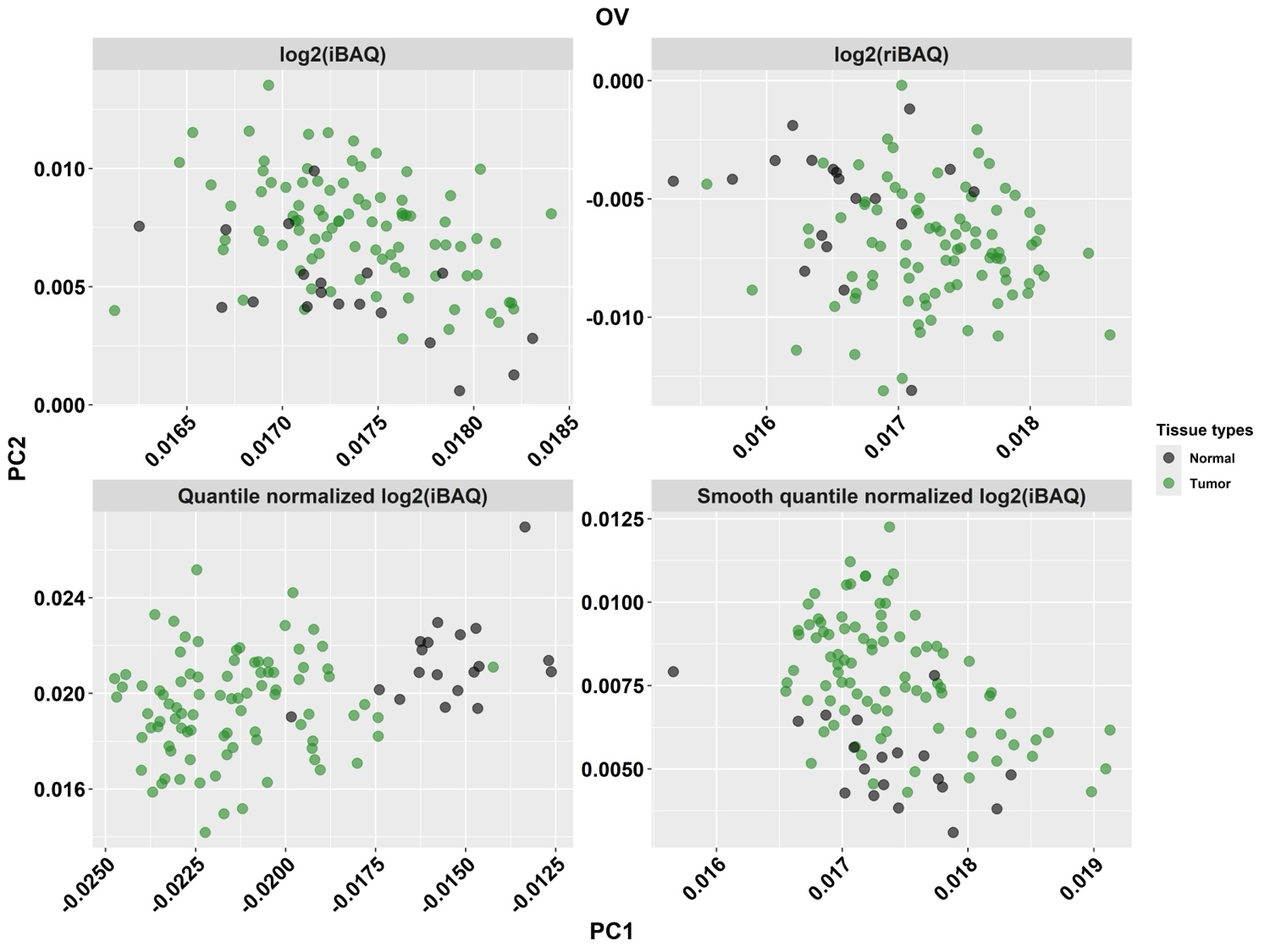


i


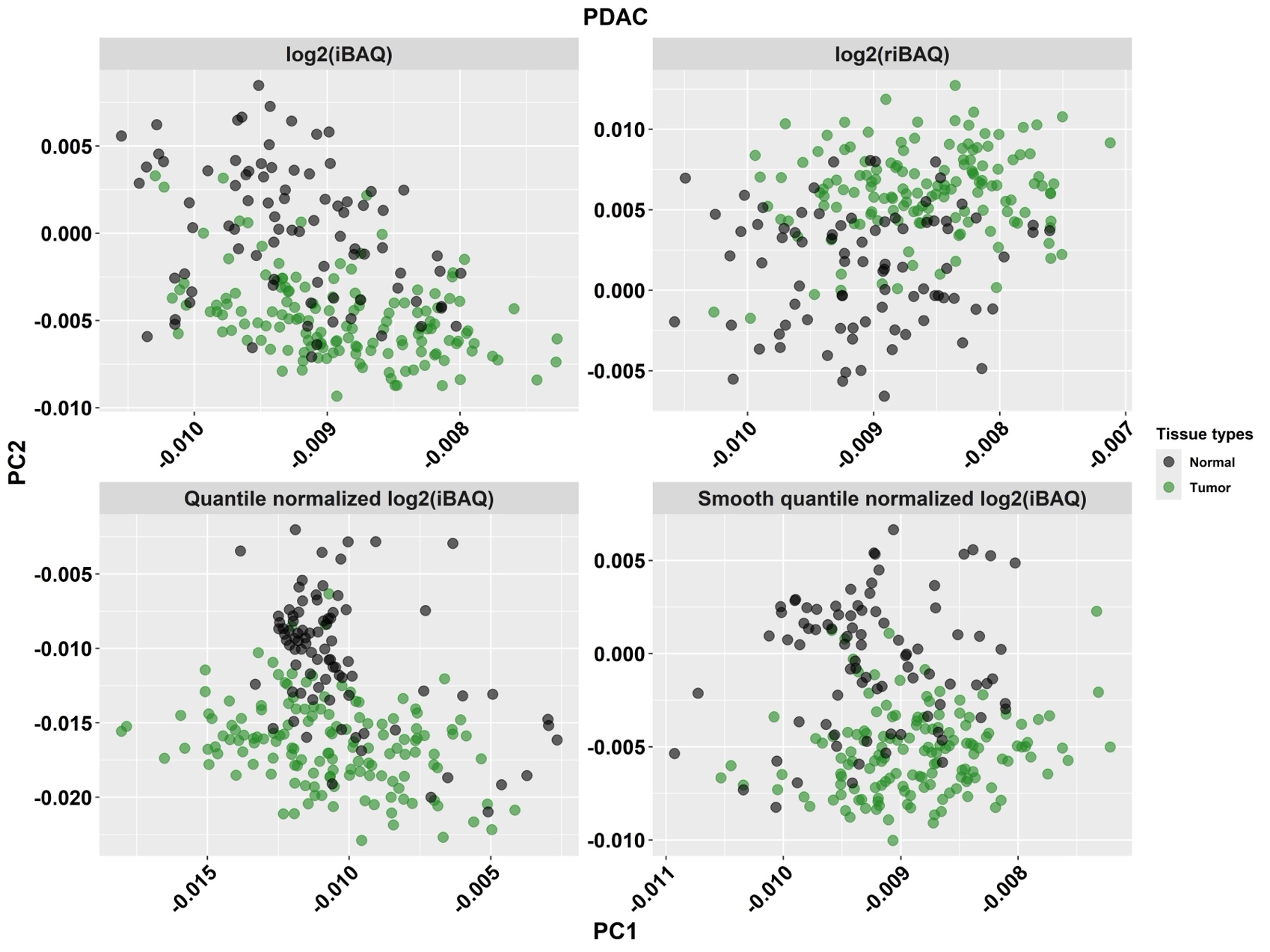


j


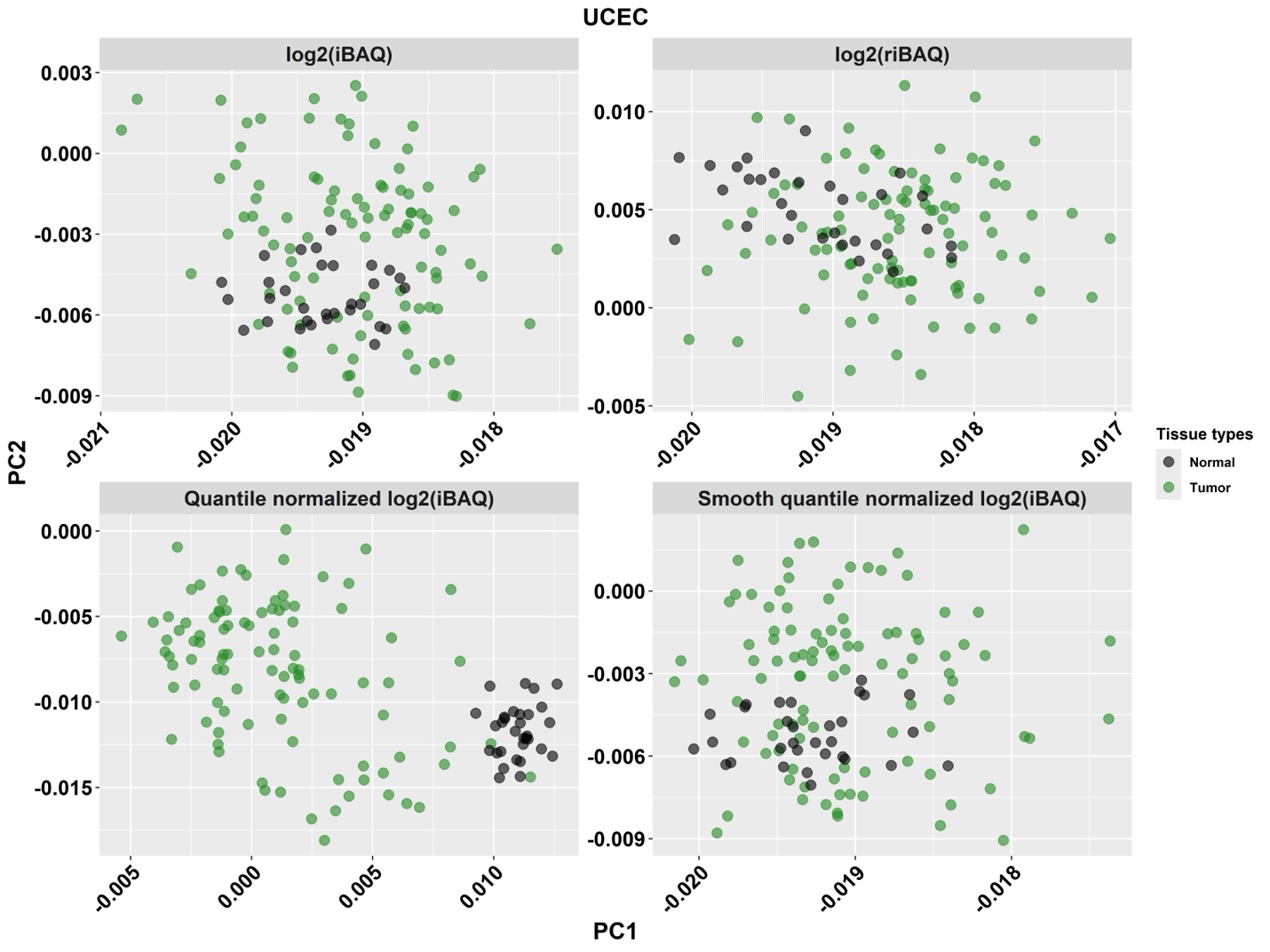


**Supplementary Figure 4.** Principal component analysis of protein expression in CPTAC indications. Data points are color coded according to tissue type. BRCA, breast cancer; ccRCC, clear-cell renal cell carcinoma; COAD, colon adenocarcinoma; GBM, glioblastoma multiforme; HNSCC, head and neck squamous-cell carcinoma; LSCC, lung squamous-cell carcinoma; LUAD, lung adenocarcinoma; OV, ovarian cancer; PDAC, pancreatic ductal adenocarcinoma; UCEC, uterine corpus endometrial carcinoma.


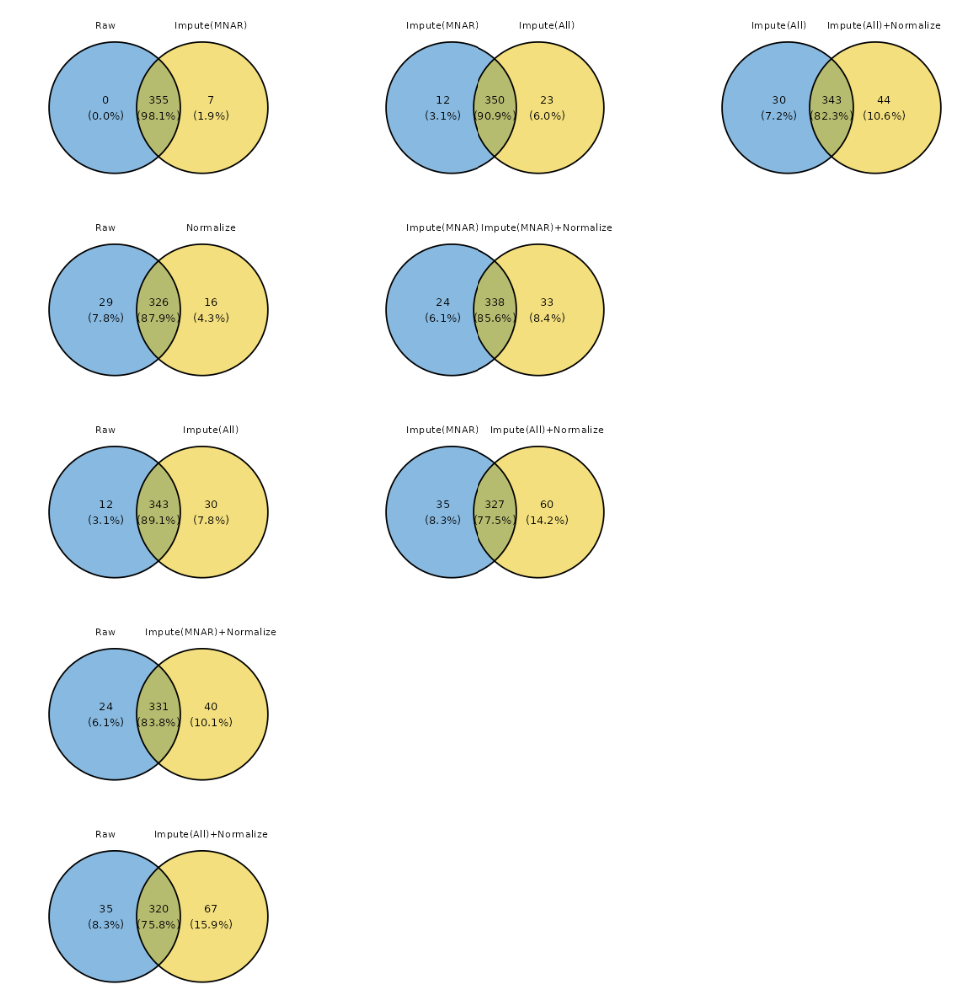


**Supplementary Figure 5.** The impact of each analytical step on the number of DEPs identified in the LUAD cohort when comparing normal and tumor tissues using raw data, imputation of only missing not at random (MNAR) values, imputation of both MNAR and missing at random (MAR) values, imputation of MNAR followed by quantile normalization, and imputation of both MNAR and MAR followed by quantile normalization.

a


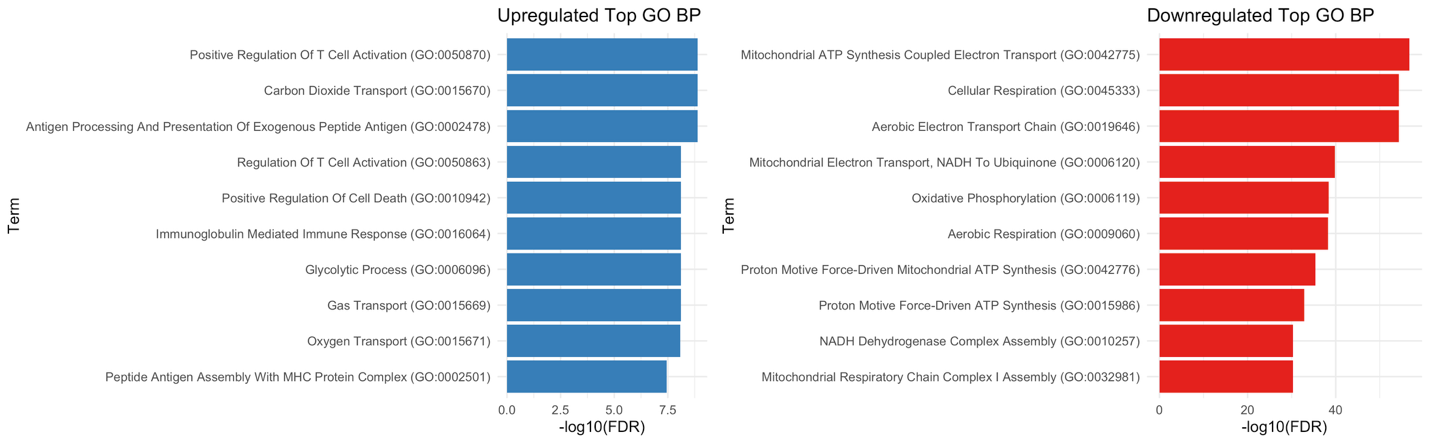


b
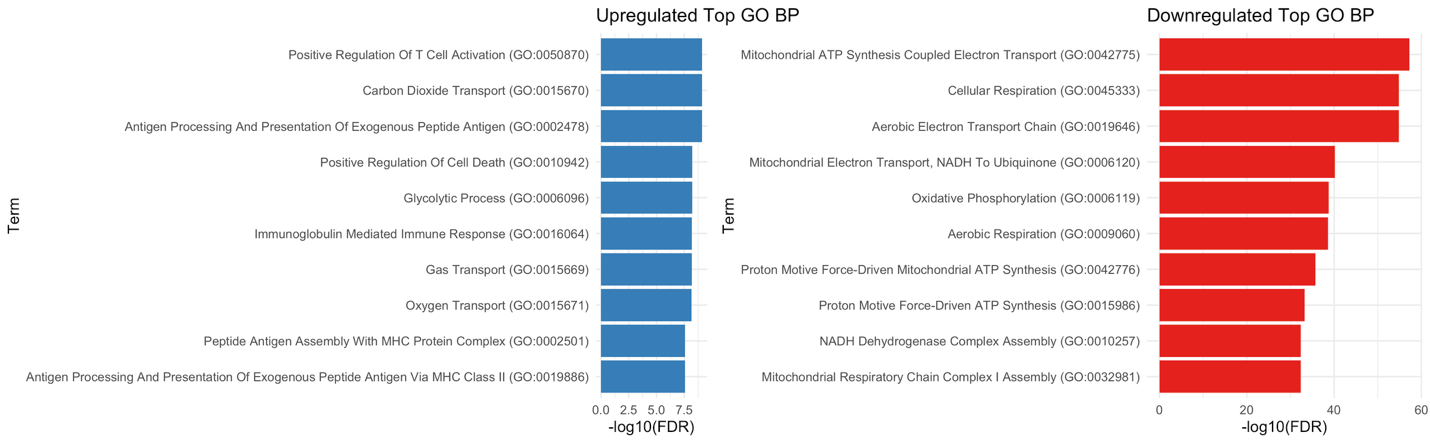


c

**
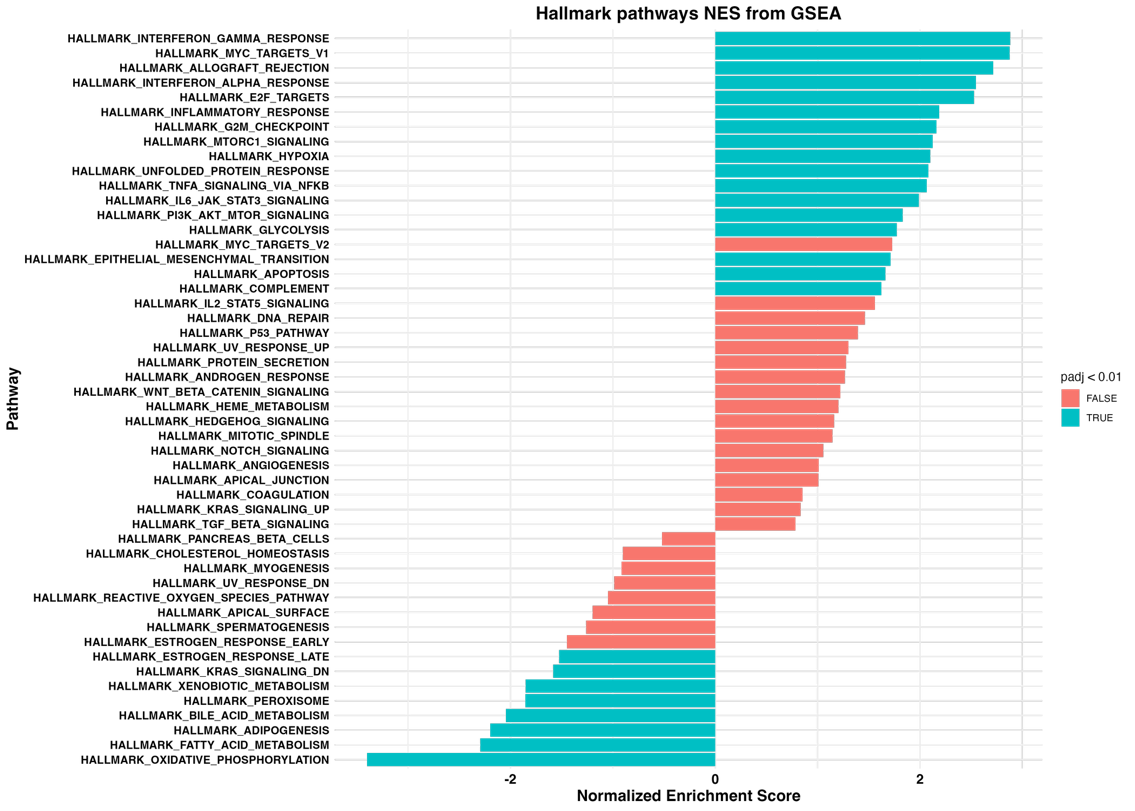
**

d

**
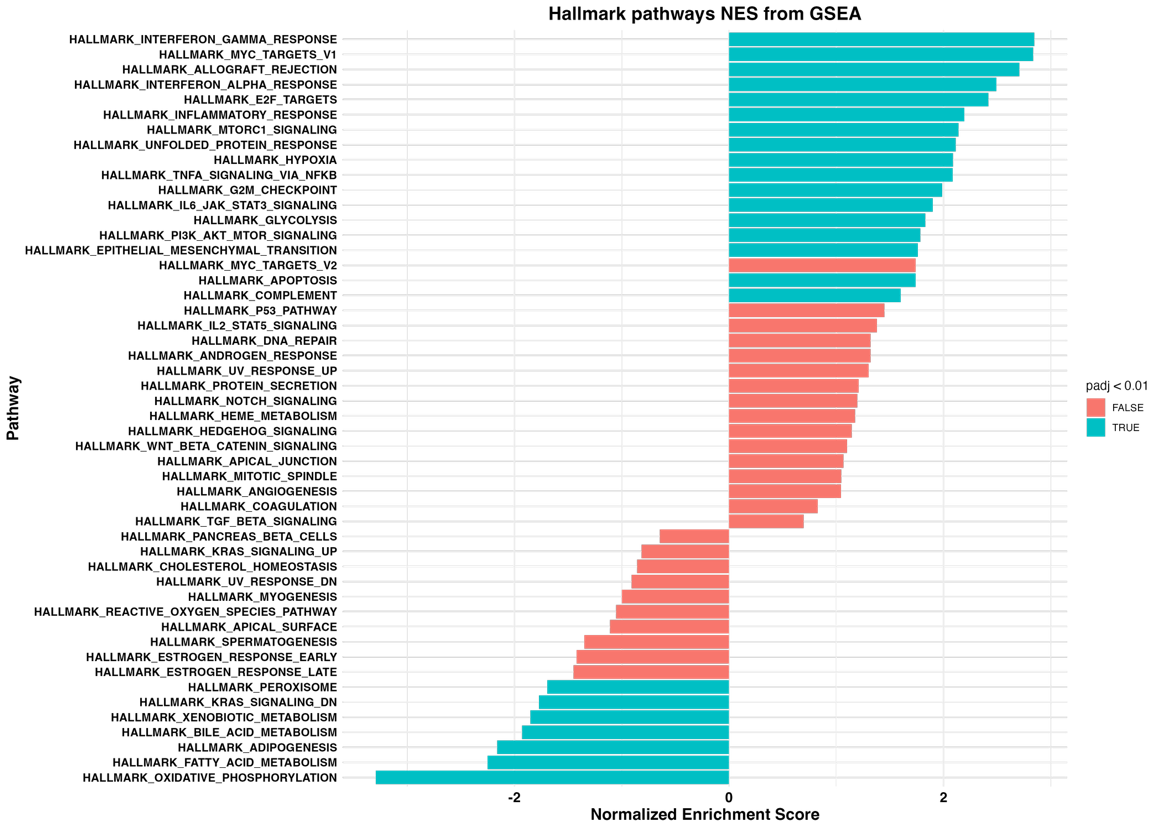
**

e.


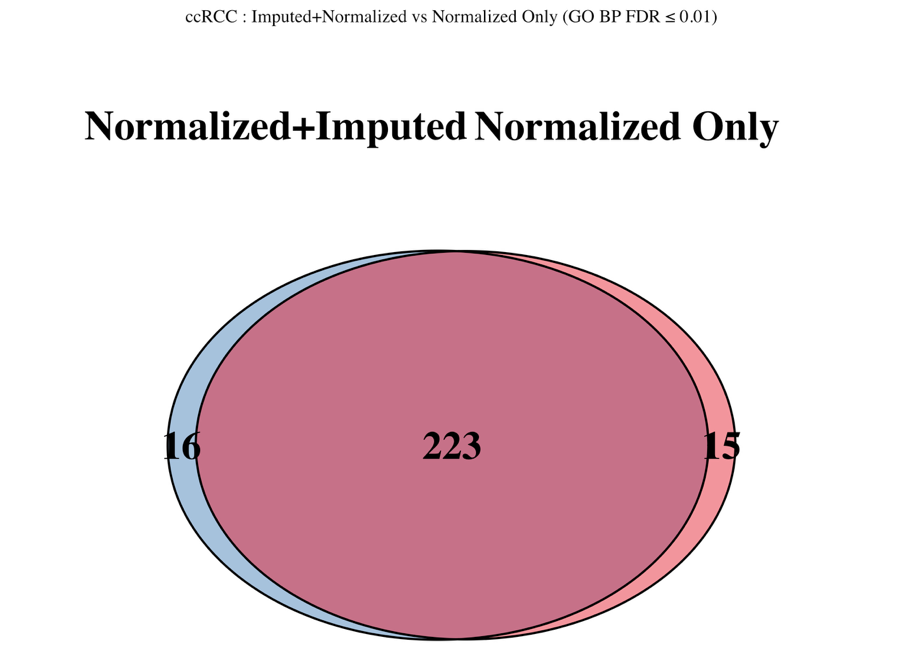


**Supplementary Figure 6.** Comparison of normalization with normalization/imputation impact on analyses for understanding transcriptional differences between tumor and normal tissues. Differential expression analysis between tumor and normal tissues for ccRCC using datasets processed through normalization alone as well as normalization combined with imputation was performed, then gene ontology enrichment analysis and gene set enrichment analysis (GSEA) was conducted. Only differentially expressed proteins with fold >= 2 and FDR <= 0.01 were used for gene ontology analysis. Gene ontology analysis was performed with enrichR package and database GO_Biological_Process_2023 was used for query. Top ten enriched GO terms with FDR <= 0.01 are shown. GO, gene ontology, BP, biological process, FDR, false discovery rate. GSEA analysis was performed with fgsea package, and MSigDB hallmark 50 gene sets were assessed. GSEA, gene set enrichment analysis. a. ccRCC top10 GO BP terms with normalized and imputed dataset b. ccRCC top10 GO BP terms with normalized only dataset, c. ccRCC GSEA MsigDB Hallmark terms with normalized and imputed dataset d. ccRCC GSEA MsigDB Hallmark terms with normalized only dataset. e. At FDR 0.01 threshold, there are 223 gene ontology biological processes are overlapped between normalized/imputed and normalized only datasets in ccRCC indication.

a


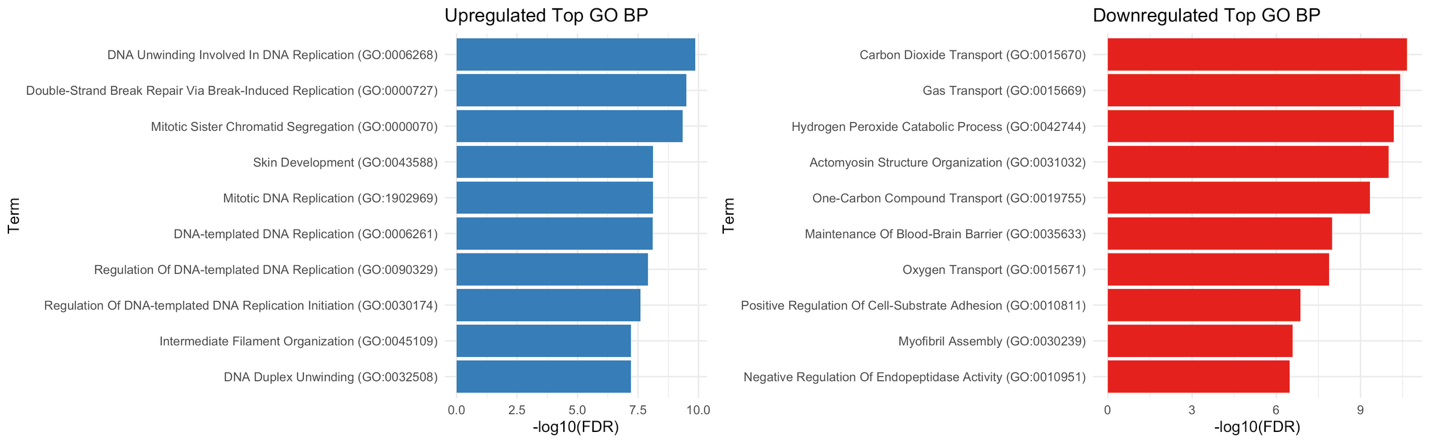


b


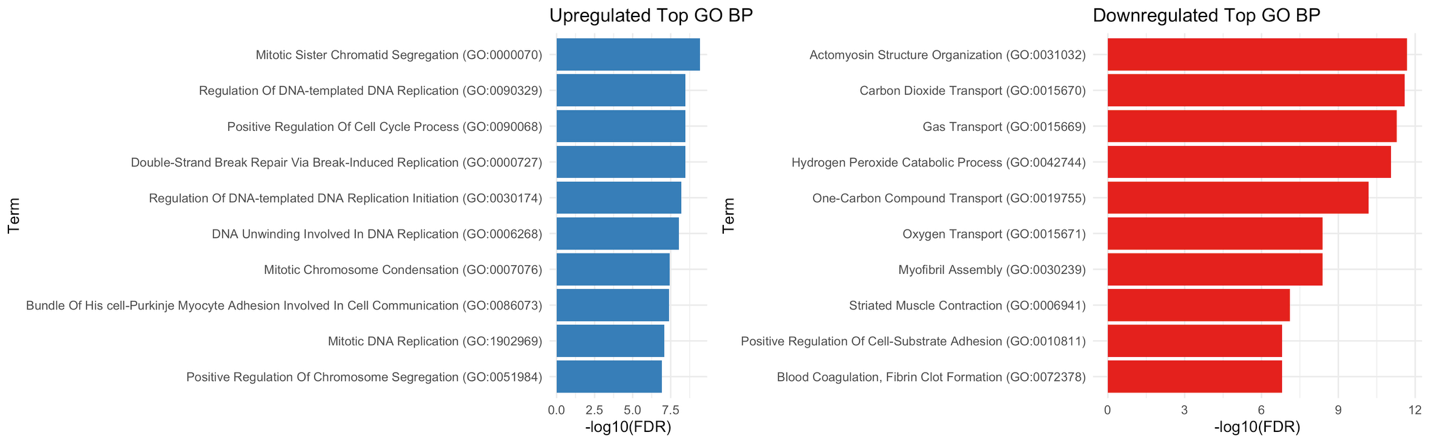


c

**
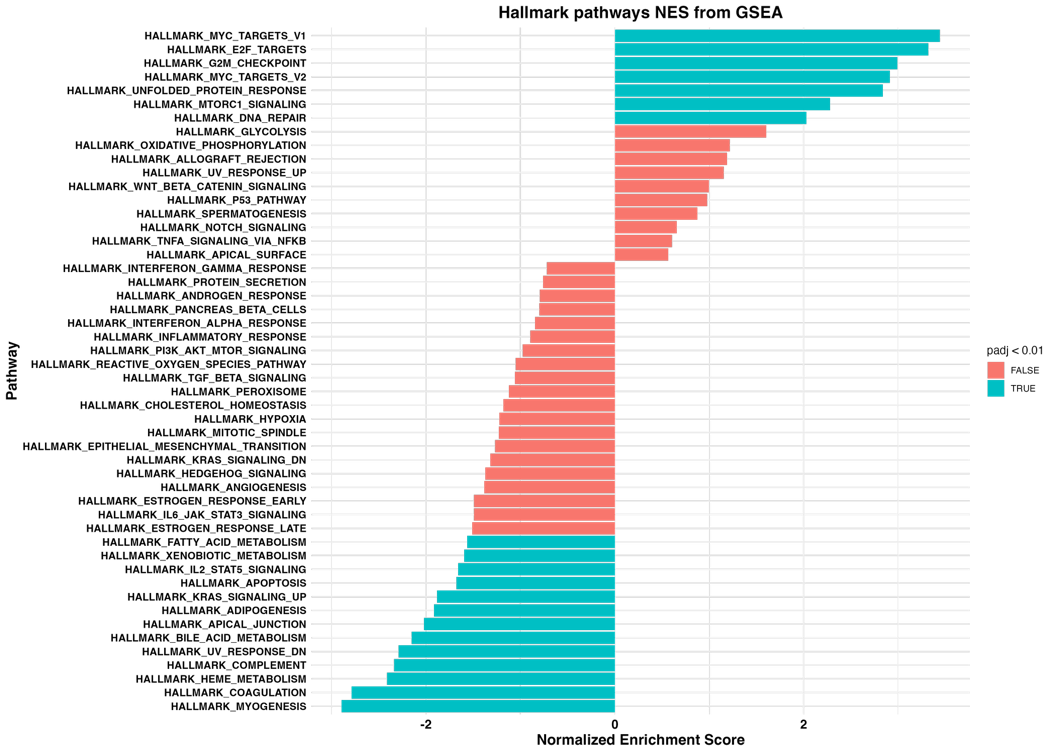
**

d

**
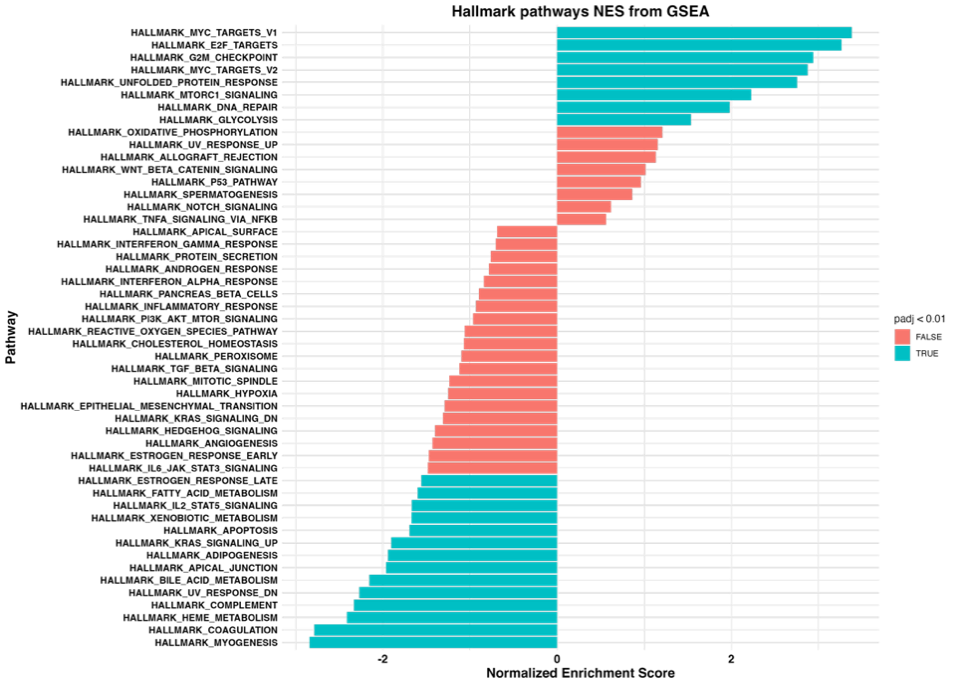
**

e.


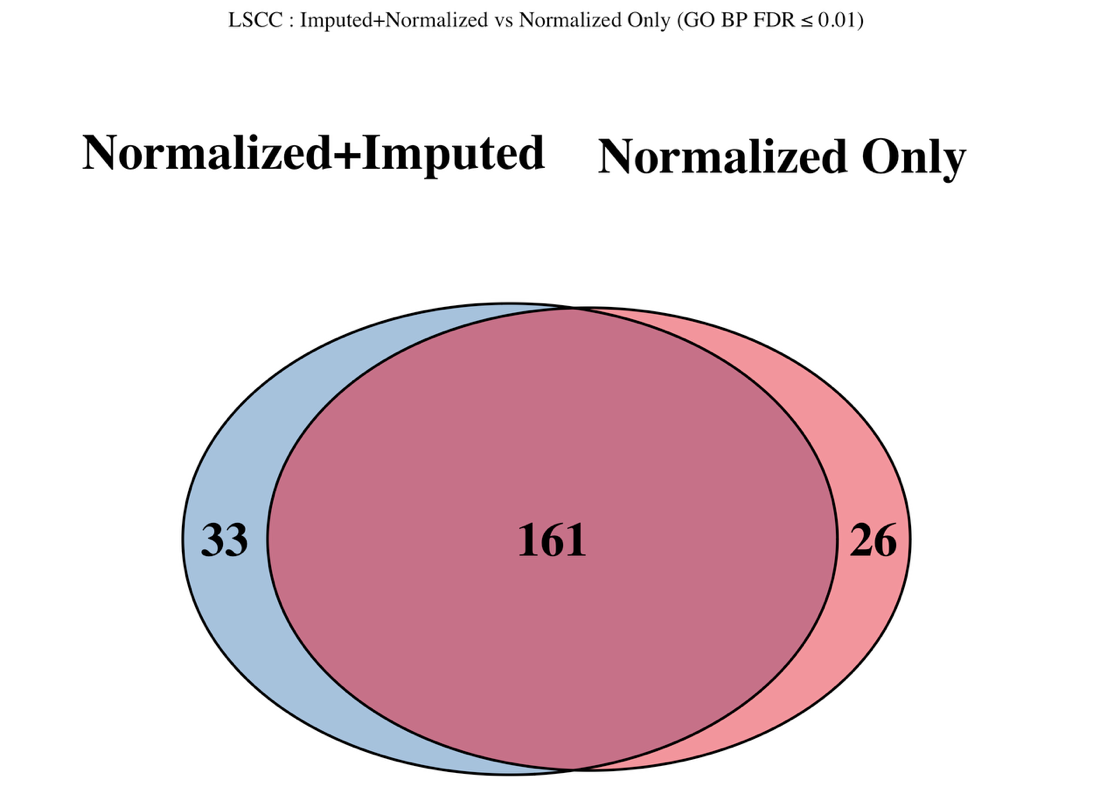


**Supplementary Figure 7.** Comparison of normalization with normalization/imputation impact on the downstream biology. Differential expression analysis between tumor and normal tissues for LSCC using datasets processed through normalization alone as well as normalization combined with imputation was performed, then gene ontology enrichment analysis and gene set enrichment analysis (GSEA) was conducted. Only differentially expressed proteins with fold >= 2 and FDR <= 0.01 were used for gene ontology analysis. Gene ontology analysis was performed with enrichR package and database GO_Biological_Process_2023 was used for query. Top ten enriched GO terms with FDR <= 0.01 are shown. GO, gene ontology, BP, biological process, FDR, false discovery rate. GSEA analysis was performed with fgsea package, and MSigDB hallmark 50 gene sets were assessed. GSEA, gene set enrichment analysis. a. LSCC top10 GO BP terms with normalized and imputed dataset b. LSCC top10 GO BP terms with normalized only dataset, c. LSCC GSEA MsigDB Hallmark terms with normalized and imputed dataset d. LSCC GSEA MsigDB Hallmark terms with normalized only dataset. e. At FDR 0.01 threshold, there are 161 gene ontology biological processes are overlapped between normalized/imputed and normalized only datasets in LSCC indication.


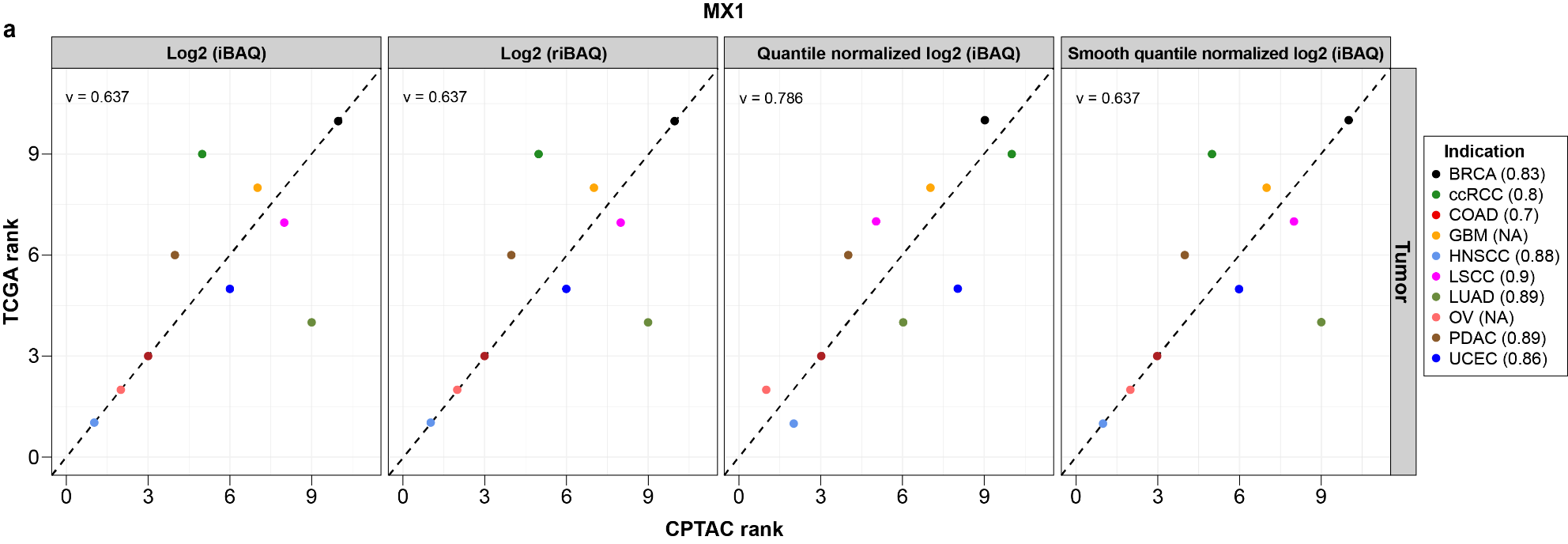


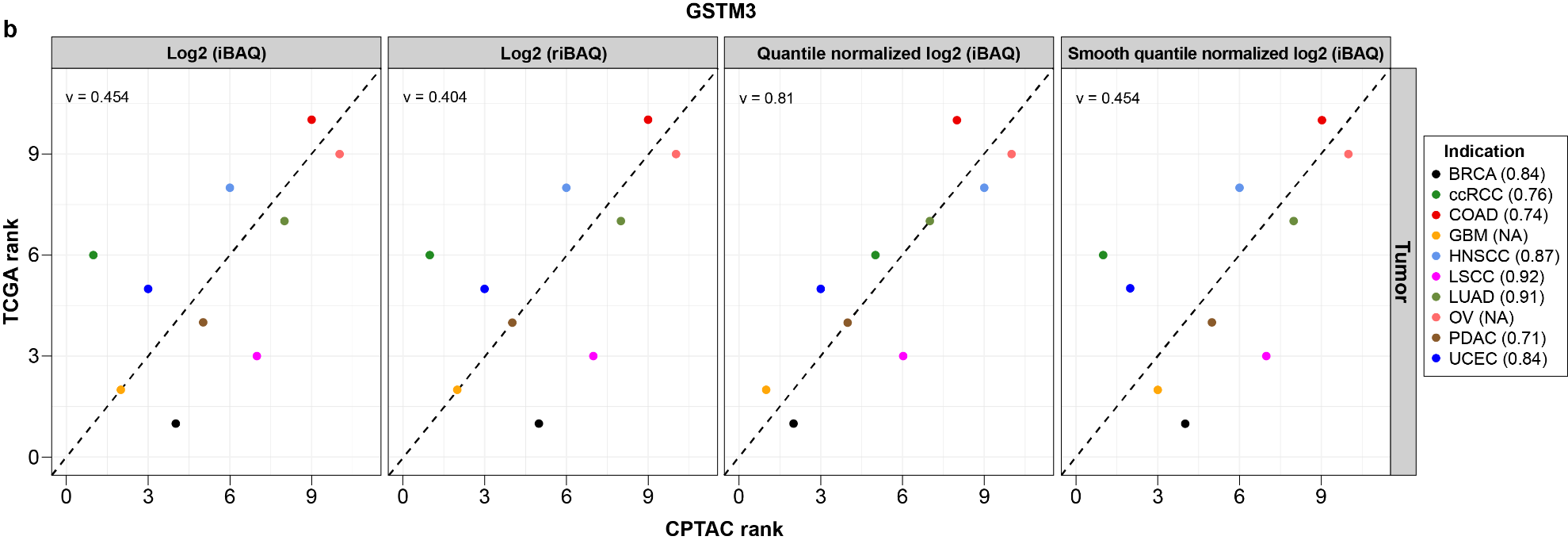


**
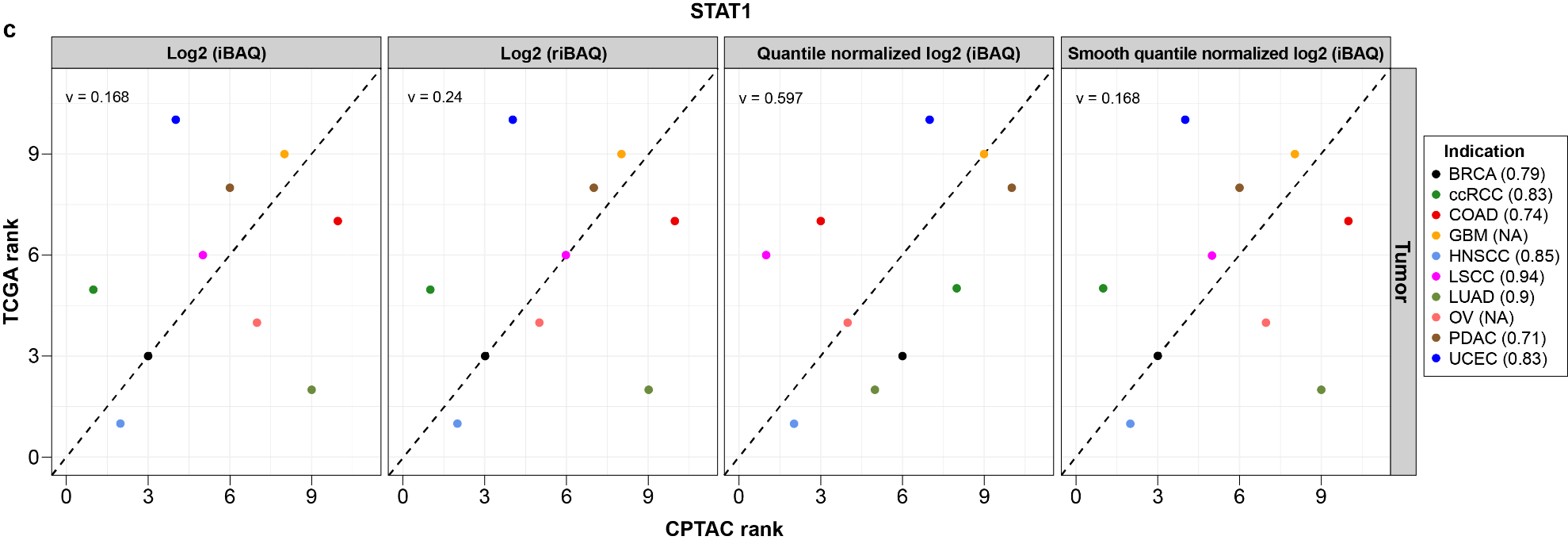
**

**
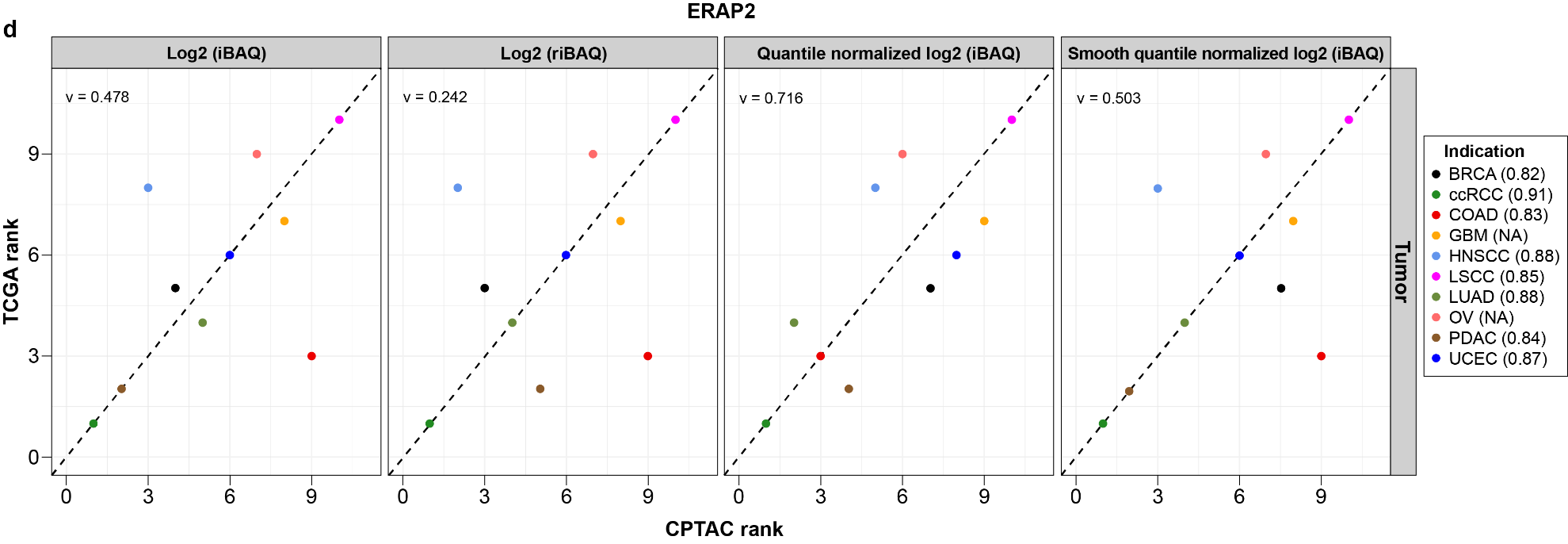
**

**
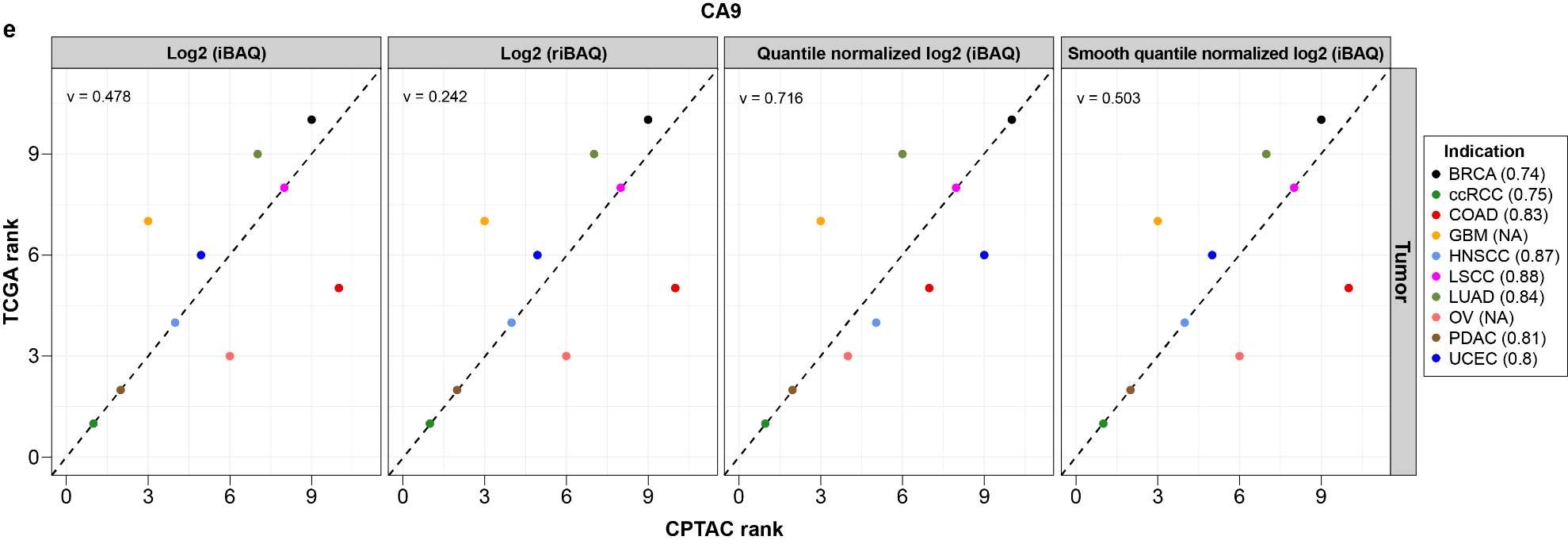
**

**Supplementary Figure 8.** Comparison of expression ranks of highly correlated proteins in CPTAC and The Cancer Genome Atlas (TCGA). Data points are color coded by indication and followed (in parentheses) by the Pearson correlation coefficient between protein and RNA expression in CPTAC. v, weighted rank correlation coefficient. Protein and RNA expression ranks from OV and GBM were plotted but excluded from the rank correlation calculation due to the limited number of proteins showing high correlation with RNA, as well as the absence of overlap with proteins selected from other indications.

**
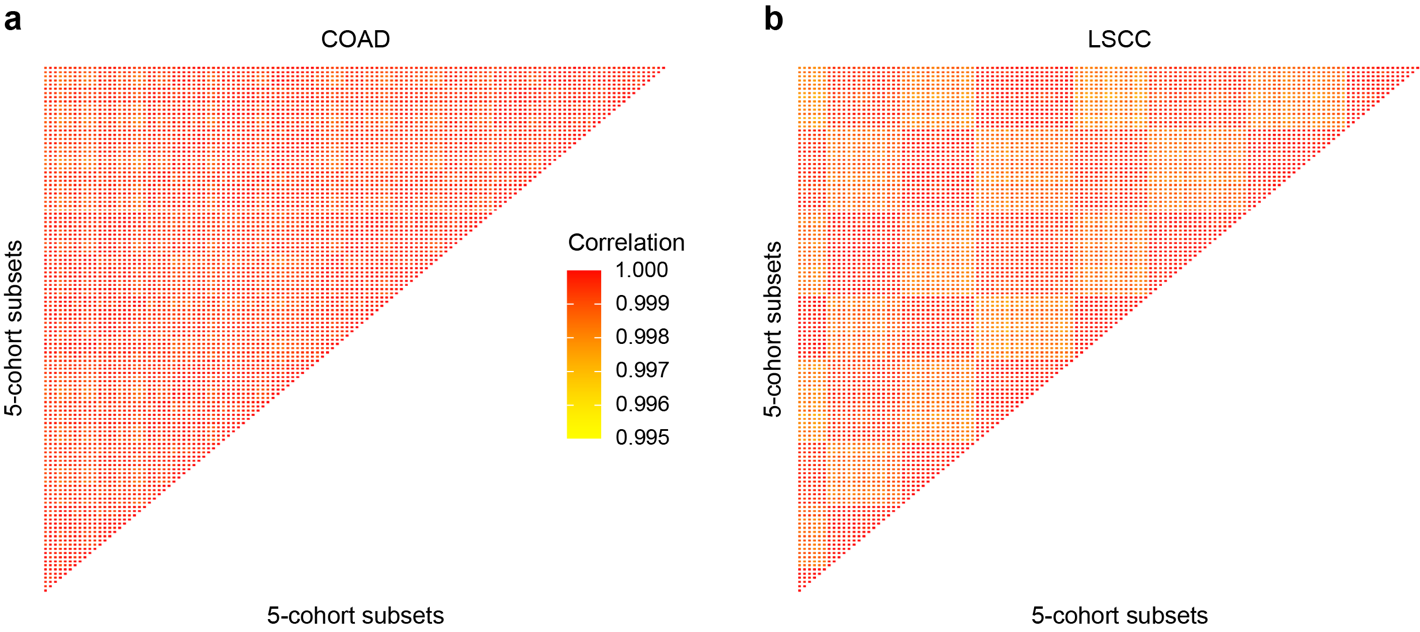
**

**Supplementary Figure 9.** Five-cohort subset analysis. Comparison of between tumor and matched normal tissues in COAD and LSCC across all relevant 5-cohort subsets.


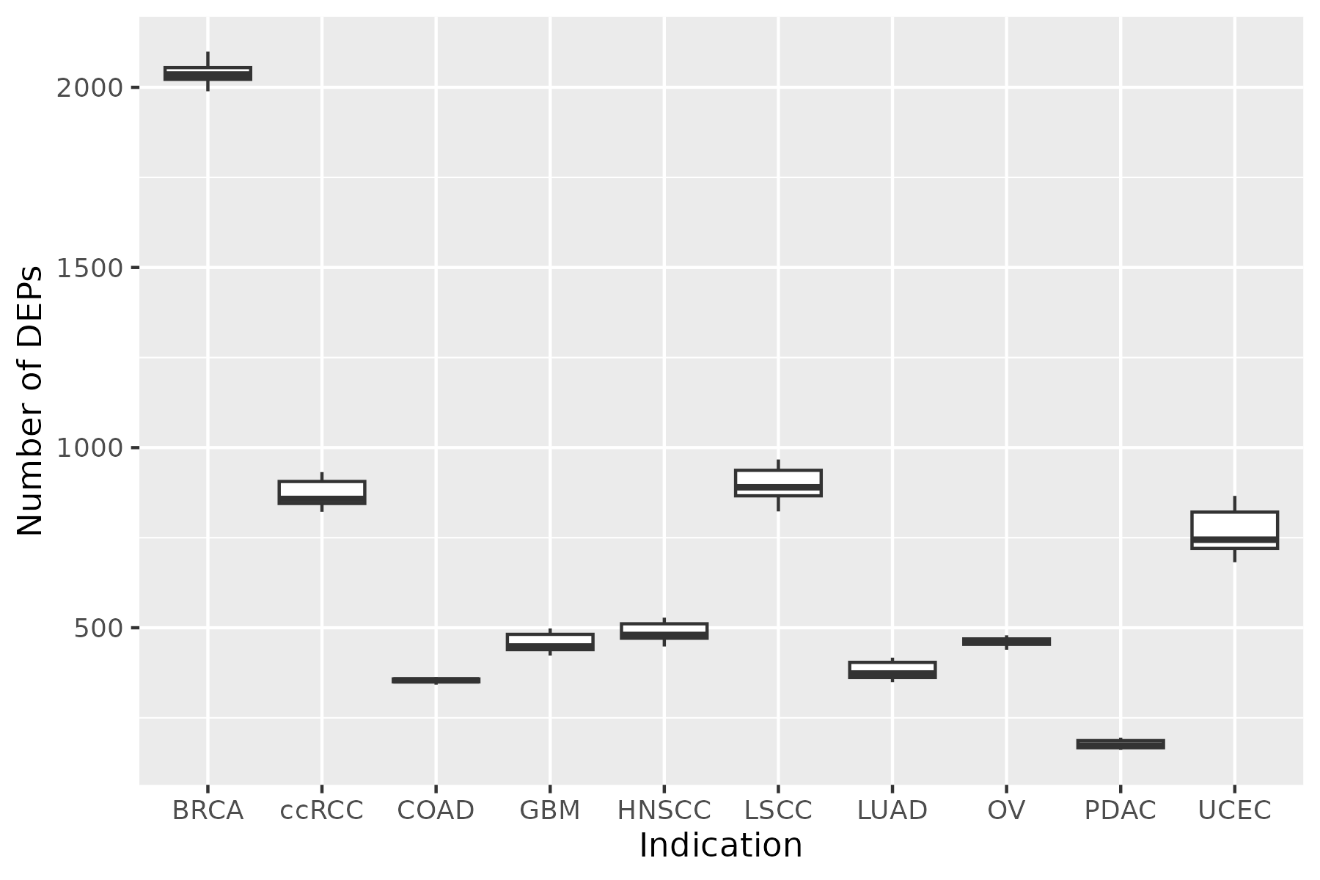


**Supplementary Figure 10.** Five-cohort subset analysis. Comparison of number of DEPs between tumor and matched normal tissues in CPTAC pan-cancer indications across all relevant 5-cohort subsets.

**Supplementary Table 1**

| **Indication** | **No. of proteins in tumor** | | **No. of tumor samples** | | **No. of proteins in normal** | | **No. of normal samples** | |
| --- | --- | --- | --- | --- | --- | --- | --- | --- |
| BRCA | 12,285 |  | 133 |  | 8989 |  | 18 |  |
| COAD | 9689 |  | 97 |  | 9689 |  | 100 |  |
| GBM | 12,694 |  | 100 |  | 12,648 |  | 10 |  |
| HNSCC | 12,457 |  | 108 |  | 12,496 |  | 58 |  |
| LSCC | 13,487 |  | 110 |  | 13,487 |  | 102 |  |
| LUAD | 12,860 |  | 119 |  | 12,864 |  | 102 |  |
| OV | 11,383 |  | 89 |  | 11,387 |  | 19 |  |
| PDAC | 12,170 |  | 145 |  | 12,170 |  | 85 |  |
| UCEC | 12,543 |  | 100 |  | 12,398 |  | 31 |  |
| ccRCC | 11,852 |  | 110 |  | 11,831 |  | 84 |  |

BRCA, breast cancer; ccRCC, clear-cell renal cell carcinoma; COAD, colon adenocarcinoma; GBM, glioblastoma multiforme; HNSCC, head and neck squamous-cell carcinoma; LSCC, lung squamous-cell carcinoma; LUAD, lung adenocarcinoma; OV, ovarian cancer; PDAC, pancreatic ductal adenocarcinoma; UCEC, uterine corpus endometrial carcinoma.

**Supplementary Table 2**

| **Indication** | **No. of proteins in tumor** | | **No. of tumor samples** | | **No. of proteins in normal** | | **No. of normal samples** | |
| --- | --- | --- | --- | --- | --- | --- | --- | --- |
| BRCA | 9587 |  | 133 |  | 8364 |  | 18 |  |
| COAD | 9065 |  | 97 |  | 9065 |  | 100 |  |
| GBM | 9581 |  | 100 |  | 9572 |  | 10 |  |
| HNSCC | 9695 |  | 108 |  | 9700 |  | 58 |  |
| LSCC | 9810 |  | 110 |  | 9810 |  | 102 |  |
| LUAD | 9686 |  | 119 |  | 9691 |  | 102 |  |
| OV | 9541 |  | 89 |  | 9544 |  | 19 |  |
| PDAC | 9684 |  | 145 |  | 9684 |  | 85 |  |
| UCEC | 9629 |  | 100 |  | 9615 |  | 31 |  |
| ccRCC | 9597 |  | 110 |  | 9592 |  | 84 |  |

BRCA, breast cancer; ccRCC, clear-cell renal cell carcinoma; COAD, colon adenocarcinoma; GBM, glioblastoma multiforme; HNSCC, head and neck squamous-cell carcinoma; LSCC, lung squamous-cell carcinoma; LUAD, lung adenocarcinoma; OV, ovarian cancer; PDAC, pancreatic ductal adenocarcinoma; UCEC, uterine corpus endometrial carcinoma.

**Supplementary Table 3.** Differential protein expression between tumor tissue and matched normal tissue in the ccRCC cohort, reported without normalization and under multiple normalization approaches. For each protein, the table lists the estimated log2 fold change, the p-value, and the Benjamini–Hochberg false discovery rate (FDR)–adjusted p-value to account for multiple testing.

**Supplementary Table 4.** Differential protein expression between tumor tissue and matched normal tissue in the COAD cohort, reported without normalization and under multiple normalization approaches. For each protein, the table lists the estimated log2 fold change, the p-value, and the Benjamini–Hochberg false discovery rate (FDR)–adjusted p-value to account for multiple testing.

**Supplementary Table 5.** Differential protein expression between tumor tissue and matched normal tissue in the LSCC cohort, reported without normalization and under multiple normalization approaches. For each protein, the table lists the estimated log2 fold change, the p-value, and the Benjamini–Hochberg false discovery rate (FDR)–adjusted p-value to account for multiple testing.

**Supplementary Table 6.** Differential protein expression between tumor tissue and matched normal tissue in the LUAD cohort, reported without normalization and under multiple normalization approaches. For each protein, the table lists the estimated log2 fold change, the p-value, and the Benjamini–Hochberg false discovery rate (FDR)–adjusted p-value to account for multiple testing.

**Supplementary Table 7.** Differential protein expression between tumor tissue and matched normal tissue in the LUAD cohort, assessing the impact of imputation and quantile normalization. Using thresholds of log2 fold change > 1 and FDR < 0.01, DEPs are identified across multiple data versions: raw data; imputed data with only missing not at random (MNAR) values imputed; imputed data with both MNAR and missing at random (MAR) values imputed; MNAR imputation followed by quantile normalization; and MNAR+MAR imputation followed by quantile normalization.

**Supplementary Table 8.** Differential expression analysis list between tumor and normal tissues using dataset processed through normalization combined with imputation in ccRCC.

**Supplementary Table 9.** Differential expression analysis list between tumor and normal tissues using dataset processed through normalization in ccRCC.

**Supplementary Table 10.** Differential expression analysis list between tumor and normal tissues using dataset processed through normalization combined with imputation in LSCC.

**Supplementary Table 11.** Differential expression analysis list between tumor and normal tissues using dataset processed through normalization in LSCC.
